# Cecum Lymph Node Dendritic Cells Harbor Slow-Growing Bacteria Phenotypically Tolerant to Antibiotic Treatment

**DOI:** 10.1371/journal.pbio.1001793

**Published:** 2014-02-18

**Authors:** Patrick Kaiser, Roland R. Regoes, Tamas Dolowschiak, Sandra Y. Wotzka, Jette Lengefeld, Emma Slack, Andrew J. Grant, Martin Ackermann, Wolf-Dietrich Hardt

**Affiliations:** 1 Institute of Microbiology, Eidgenössische Technische Hochschule ETH, Zurich, Switzerland; 2 Institute of Integrative Biology, Eidgenössische Technische Hochschule ETH, Zurich, Switzerland; 3 Department of Veterinary Medicine and Cambridge Infectious Diseases Consortium, University of Cambridge, Cambridge, United Kingdom; 4 Department of Environmental Systems Science, ETH Zurich, and Department of Environmental Microbiology, Eawag, Switzerland; Stanford University, United States of America

## Abstract

In vivo, antibiotics are often much less efficient than ex vivo and relapses can occur. The reasons for poor in vivo activity are still not completely understood. We have studied the fluoroquinolone antibiotic ciprofloxacin in an animal model for complicated Salmonellosis. High-dose ciprofloxacin treatment efficiently reduced pathogen loads in feces and most organs. However, the cecum draining lymph node (cLN), the gut tissue, and the spleen retained surviving bacteria. In cLN, approximately 10%–20% of the bacteria remained viable. These phenotypically tolerant bacteria lodged mostly within CD103^+^CX_3_CR1^−^CD11c^+^ dendritic cells, remained genetically susceptible to ciprofloxacin, were sufficient to reinitiate infection after the end of the therapy, and displayed an extremely slow growth rate, as shown by mathematical analysis of infections with mixed inocula and segregative plasmid experiments. The slow growth was sufficient to explain recalcitrance to antibiotics treatment. Therefore, slow-growing antibiotic-tolerant bacteria lodged within dendritic cells can explain poor in vivo antibiotic activity and relapse. Administration of LPS or CpG, known elicitors of innate immune defense, reduced the loads of tolerant bacteria. Thus, manipulating innate immunity may augment the in vivo activity of antibiotics.

## Abstract 2

*Salmonella* bacteria can tolerate antibiotics by adopting a slow-growing “persister” state that hides in host dendritic cells and can re-initiate infection after treatment ends. This can be avoided by supplementing antibiotic treatment with stimulants of innate immunity.

## Author Summary

Antibiotics that are known to kill bacteria in vitro can be less efficacious in vivo. The reasons for this have remained poorly understood. Using a mouse model for *Salmonella* diarrhea, we found that bacterial persistence occurs in the presence of the antibiotic ciprofloxacin because *Salmonella* can exist in two different states: as a fast-growing population that spreads in the host's tissues and as a slow-growing “persister” subpopulation. The slow-growing bacteria infect and hide out inside dendritic cells of the host's immune system and cannot be attacked by the antibiotic—they are thereby rendered “tolerant,” despite their genetic susceptibility to the drug. These tolerant bacteria form a reservoir of viable cells that are able to reinitiate the infection on cessation of antibiotic therapy. Fortunately, however, these tolerant *Salmonella* cells are not invincible, and can be killed by adding agents that directly stimulate the host's immune defense. Combining innate immune stimulants with antibiotic treatment may offer new opportunities to improve antibacterial therapies.

## Introduction

Antibiotics are of great importance for treating bacterial infections. However, the resistance of bacteria against antibiotics has remained a significant problem of global concern [1]. Resistance can be conferred by resistance determinants encoded in the pathogen's genome, as well as by “noninherited resistance” (also termed “phenotypic tolerance” or “persistence”; [2]). Such tolerance is a phenotypic adaptation allowing survival of genotypically susceptible bacteria at antibiotic concentrations exceeding the “minimal inhibitory concentration” (MIC). Although the molecular basis of phenotypic tolerance is still not entirely clear, the bacterial growth rate is often a cardinal factor [3]. Most (if not all) genetically susceptible bacteria are exquisitely susceptible during exponential growth, but display tolerance against diverse classes of antibiotics in the stationary phase [2],[4]. Early hints and a growing body of anecdotal observations suggest that slow pathogen growth rates in vivo may explain why antibiotics therapy in vivo takes much longer and is much less efficient than predicted from ex vivo analysis of exponentially grown cultures [5]–[7]. To verify this hypothesis, we would need robust experimental systems quantifying the growth rates of tolerant bacteria in vivo.

To study bacterial tolerance to antibiotics in vivo, we have chosen the pathogenic bacterium *Salmonella enterica* serovar Typhimurium (*S.* Tm). In humans, the majority of cases develop “noncomplicated,” self-limiting diarrhea, where the pathogen remains restricted to the gut lumen, gut tissue, and the gut-associated lymphatic tissue. However, in complicated cases (i.e., young children, elderly, immunocompromised patients), *S.* Tm spreads beyond the gut-draining lymph nodes and to systemic sites, thus causing a life-threatening infection. In these cases, antibiotics (e.g., fluoroquinolones like ciprofloxacin; typically two doses of approximately 7 mg/kg per day) are used for therapy [8],[9]. Fluoroquinolones are broad-spectrum gyrase inhibitors, interfere with bacterial DNA replication, enhance bacterial DNA fragmentation, and display bactericidal activity against many Gram-negative and Gram-positive bacteria [10]. However, in spite of exquisite in vitro activity (within minutes to hours), and excellent tissue penetration characteristics of fluoroquinolones [11], the in vivo activity is generally much lower, requiring treatment for at least 5–10 d with frequent relapses [12], increased risks of long-term carriage [8], and long-term persistence of the pathogen in blood and bone marrow [13]. Before the introduction of efficient antiretroviral therapy, AIDS patients displayed high susceptibility to complicated *S.* Typhimurium infection. Antibiotic therapy did relieve the symptoms. However, due to high rates of relapse, many AIDS patients underwent life-long antibiotic therapy ([14],[15]). Intriguingly, the bacteria re-isolated from relapses do generally remain genetically susceptible to the respective antibiotic. Thus, tolerance might play a significant role. It has remained enigmatic why the pathogen is tolerant to antibiotic therapy in vivo, how this may impact the pathogen–host interaction, and how tolerant bacteria can be eliminated.

## Results

We analyzed antibiotic tolerance of *S.* Tm in vivo in an intragastric mouse infection model using C57BL/6 mice, which are susceptible to enteropathy and systemic spread of the pathogen [16]. This model displays the typical features of complicated Salmonellosis—that is, diarrhea accompanied by pathogen spread to the cecal lymph node (cLN) and systemic sites. Without antibiotic treatment, the mice would succumb to systemic infection by wild-type *S.* Typhimurium within 5 to 6 d. In this model, therapy with two doses of 15 mg/kg ciprofloxacin per day is known to reduce fecal pathogen shedding and systemic pathogen loads. However, viable pathogens could be recovered from the cLN and relapse occurs at high frequency soon after the discontinuation of the therapy [17].

To establish the kinetics of pathogen clearance by ciprofloxacin, we infected mice with *S.* Tm (strain SL1344, 5×10^7^ cfu by gavage) and began high-dose ciprofloxacin treatment from day one postinfection (2×62 mg/kg per day by gavage). This high dosage of ciprofloxacin was chosen to achieve systemic antibiotic concentrations of ≥50-fold the MIC throughout the duration of the experiment (MIC≤0.03 µg/ml as determined by the in vitro plating assay; Table S1 and Figure S1) and to ensure pathogen clearance from the gut lumen of all mice (in spite of reduced gut luminal bioavailability—e.g., through antibiotic absorption by food particles). This allowed us to focus on the tolerant bacteria in the host tissue.

**Figure 1:**
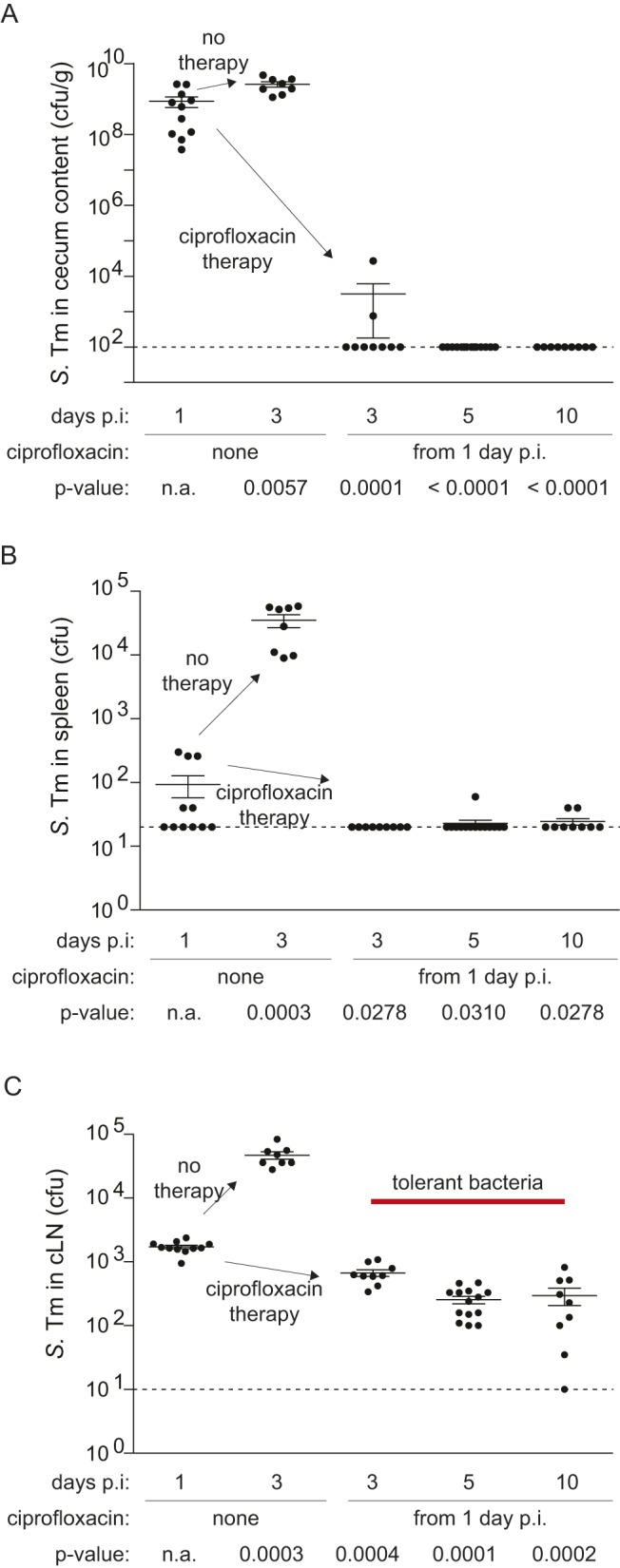
Ciprofloxacin treatment cannot clear *S.* Tm from the cLN. Cfus in the cLN of C57BL/6 mice 1 d and 3 d postinfection without therapy or 3 d, 5 d, and 10 d p.i. with ciprofloxacin therapy (2×62 mg/kg/d by gavage) starting 1 d p.i. Each symbol represents one mouse. (A), cfu in stool from cecum lumen; (B), cfu in spleen; (C), cfu in cLN; dashed line, detection limit. *p*, nonparametric statistical analysis compared to 1 d p.i.

Within 3 h of ciprofloxacin treatment, the pathogen was cleared from the gut lumen and pathogen spread to the spleen was prevented by the antibiotic (Figure 1A,B and Figure S2A). In contrast, ciprofloxacin did not clear the pathogen from the gut-draining cLN (Figure S2B). cLN-loads declined with fast kinetics by 10-fold during the first 2 h after ciprofloxacin administration. This indicated that ciprofloxacin is bio-active at this site. After the first 2 h, the killing kinetics slowed down and 50–1,000 viable bacteria remained for up to 10 d (Figure 1C, Figure S2B). Such bi-phasic killing is a typical feature if pathogens form a tolerant subpopulation [3]. Indeed, bi-phasic killing is also observed if *S.* Tm bacteria taken from the logarithmic growth phase are exposed to ciprofloxacin (Figure S2C). However, in this case, only <0.1% of the bacteria survived after 6 h. This provided a first hint that pathogen–host interactions in vivo might affect poor pathogen elimination from the cLN (Figure 1C).

Subsequent experiments established that the cecal gut tissue and the cecal patch may also harbor *S.* Typhimurium cells surviving the antibiotic treatment (Figure S3). Please note that these numbers have to be interpreted with caution, as gut luminal contamination [i.e., from relapses (see, below) or bacteria closely associated with the mucosal surface] cannot be completely ruled out. We also observed some antibiotic-tolerant bacteria in the spleens at 10 d after infection or even earlier if the ciprofloxacin treatment was started on days 2 or 3 postinfection (Figure S4). In that case, the starting loads in the spleen were equivalent, but the fraction of surviving *S.* Typhimurium cells was much lower than in the cLN (0.5%–5% as compared to ∼20%; compare Figure S4B with Figure S4A, Figure 1C). These data established that *S.* Typhimurium cells can survive antibiotic treatment at several sites within the mouse. The cLN was chosen for subsequent analyses, as this site harbors a high fraction of surviving bacteria, is easily retrievable without the risk of contamination, and allows fast and efficient tissue dissociation for FACS.

In order to verify that the surviving *S.* Typhimurium cells were indeed “tolerant,” we have performed a number of control experiments. Pharmacokinetic analysis showed that the ciprofloxacin concentration always remained ≥50-fold above the minimal concentration required for inhibiting *Salmonella* growth ex vivo (MIC<0.03 µg/ml; Table S1 and Figure S1). Furthermore, tissue culture infection experiments and confocal fluorescence microscopy verified that ciprofloxacin is indeed efficiently penetrating into infected host cells (Figure S5). In line with the fast initial killing rate in the cLN of an antibiotic-treated mouse, these data verified a high bioavailability of ciprofloxacin in the tissues of interest. Furthermore, pathogen survival was not attributable to genetic changes, as all re-isolated colonies tested remained ciprofloxacin sensitive (same MIC as parental strain; Table S1) and fully virulent when transferred into naïve hosts (Figure S6). Thus, the cLN provides an environment that selects for or induces antibiotic-tolerant bacteria. Tolerant bacteria were observed in the cLN no matter when the treatment was started (Figure S4), or which mouse line or pathogen strain was used (Figure S7, *nramp*-positive 129SvEv mice; Figure S8, *S.* Tm ATCC14028). Thus, in our mouse model, the cLN are a site harboring phenotypically tolerant *S.* Tm. However, it had remained unclear if these bacteria were capable of causing a relapse, as observed a few days after the end of such an antibiotic treatment [17].

**Figure 2:**
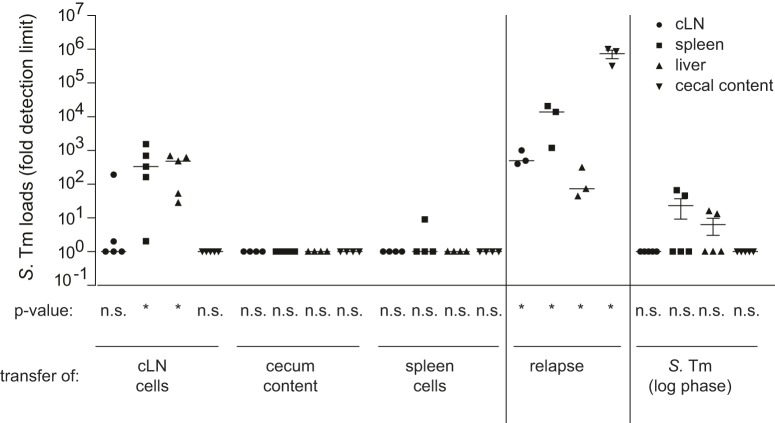
*S.* Tm in cLN from ciprofloxacin-treated mice are sufficient for initiating an infection. C57BL/6 mice were infected for 1 d with *S.* Tm before ciprofloxacin therapy was started (2×62 mg/kg/d by gavage). Recipient mice remained uninfected and were also treated with ciprofloxacin. (Left) 3 d p.i. mice were sacrificed and single-cell suspensions of the cLN or spleen or cecal content were transferred into antibiotic-treated naïve recipient mice. Four days later, recipient mice were sacrificed and pathogen loads in the respective organs were determined. Transfer of cLN cells leads to significantly higher infection rates compared to transferred spleen cells, cecum content, or *S.* Tm cultured in LB (right part; Fisher's exact test, *p* = 0.00482). (Middle) Relapse control. Mice were infected for 1 d with *S.* Tm, treated with ciprofloxacin for 2 d, and left untreated for 4 additional days. Then, we analyzed pathogen loads in the cecum content, cLN, spleen, and liver.

The capacity of tolerant bacteria from the cLN to cause a relapse-like infection was analyzed in cell transfer experiments. We have chosen the cLN population for this experiment, because it harbors a high density of tolerant cells and allows cell retrieval with minimal risk of contamination (e.g., from gut luminal contents). This cell transfer technique allowed us to focus on the bacteria located within the cLN, as all pathogens detected in the recipient animal must originate from this organ, while contributions of other bacterial populations (e.g., gut mucosa, cecal patch, spleen, or other, nonidentified organs), which might serve as additional reservoirs of tolerant bacteria, could be excluded. Donor mice (C57BL/6) were infected with *S.* Tm as described in Figure 1, while the acceptor mice remained uninfected during this initial phase of the experiment. One day later, high-dose ciprofloxacin therapy was applied to both groups. After 2 d of therapy, the infected mice were sacrificed and we recovered the cecum content, the cLN, and the spleens. Single cell suspensions were prepared from each sample and pathogen loads were verified by plating an aliquot. In line with the findings above, no bacteria were detected in the cecum contents or the spleens of the donor mice, while a total of ∼1,000 bacteria had survived therapy in the cLN. The remainder of the cell suspension was injected into the peritoneal cavity of noninfected recipient mice. As a control for viable, but nontolerant bacteria, we infected a fourth group of mice with *S.* Tm (500 cfu i.p.) from an early log-phase culture. These bacteria should be highly susceptible to ciprofloxacin levels present in the recipient mice (Figure S1, Figure S9; [18]). After the cell transfer, the ciprofloxacin treatment of the recipients was discontinued so that antibiotic concentrations drop gradually (Figure S1). Four days later, recipient mice were sacrificed and pathogen loads in the respective organs were determined. Transfer of cLN cells leads to significantly higher infection rates compared to transferred spleen cells, cecum content, or *S.* Tm cultured in LB (Fisher's exact test: *p* = 0.0048; Figure 2, left part; Figure S10). This is consistent with the high fraction of tolerant *S.* Typhimurium cells observed in the cLN (Figure 1). The loads in spleens and livers of the recipient mice were equivalent to those observed upon discontinuation of ciprofloxacin treatment in the donor mice (“relapse control”; Figure 2, right part; Figure S10; [17]). In contrast, cLN and gut luminal colonization were much lower in the recipient animals than in the relapse control. This may suggest that the site of re-seeding affects the colonization patterns during a relapse. In conclusion, the cell transfer experiments verified that the tolerant pathogens surviving in the cLN are indeed capable of re-initiating infection and can therefore account for relapses. However, it remained unclear whether the in vivo tolerance was linked to a particular intracellular niche of the cLN.

**Figure 3:**
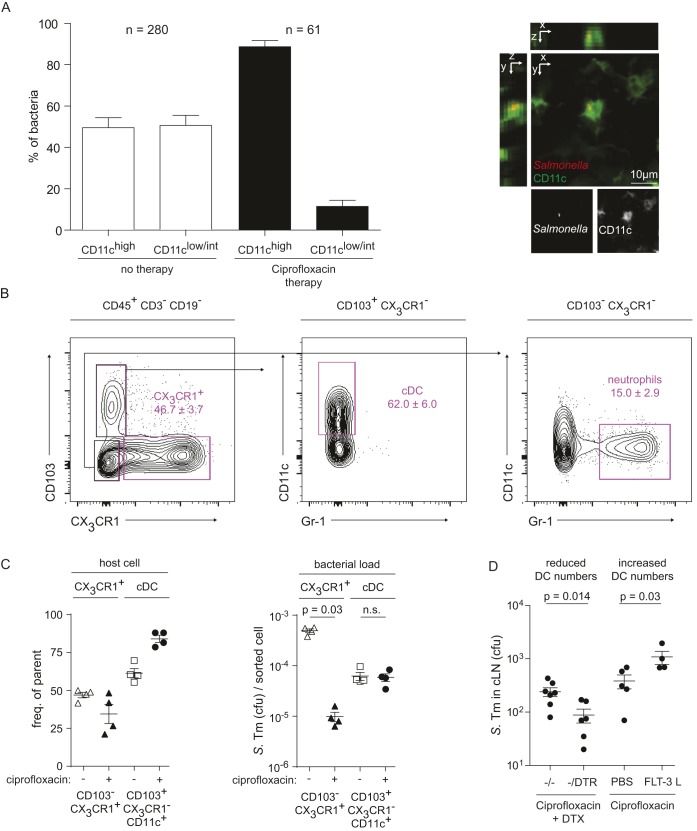
Ciprofloxacin-tolerant bacteria reside within classical CD11c^+^ dendritic cells of the cLN. (A) CD11c-YFP mice were infected with *S.* Tm pDsRed and subjected to ciprofloxacin therapy (2×62 mg/kg/d by gavage; control group was treated with PBS). We prepared 20 µm cryo sections of the cLNs and analyzed them by fluorescence microscopy. Quantification shows accumulation of bacteria within CD11c^high^ cells. (B) Gating approach used for subsequent sorting by flow cytometry. Three nonlymphoid subsets were defined as (1) CX_3_CR1^+^ iDCs: CD103^−^CX_3_CR1^+^, (2) “classical” dendritic cells CD103^+^CX_3_CR1^−^CD11c^+^Gr-1^−^ (cDC), and (3) CD103^−^CX_3_CR1^−^CD11c^−^Gr-1^+^ “neutrophils.” (C) Frequency and respective bacterial load of *S.* Tm harboring CX_3_CR1^+^ interstitial and classical DC subsets. Filled symbols, ciprofloxacin therapy; empty symbols, no therapy. (D) Depletion of CD11c^+^ cells in CD11c-DTR mice via DTX reduces the loads of tolerant bacteria (left side), whereas expansion of CD11c^+^ cells via FLT3-L injection (10 µg/mouse/d for 3 d) increases the loads of tolerant bacteria in the cLN of ciprofloxacin-treated mice (right side).

To identify cellular niches harboring the antibiotic tolerant *S.* Tm, we performed microscopy and FACS. Initially, we focused on dendritic cells, as these are important host cells during intestinal infection that can engulf and transport viable bacteria within the gut and the associated lymphatic tissue [19]–[25]. CD11c-YFP mice [26], which express a fluorescent marker protein in dendritic cells, were infected with *S.* Tm (pRFP; red fluorescence) for 1 d and treated (or not) with ciprofloxacin. In the cLN of untreated mice, the bacteria were located within dendritic cells (CD11c^+^; 53%) and other cells (CD11c^−^; 47%), as detected by fluorescence microscopy. Upon ciprofloxacin therapy, most remaining bacteria were located within CD11c^+^ cells (>80%; Figure 3A). It should be noted that this method did not allow us to establish if all detected bacteria were indeed still alive. Nevertheless, these data suggested that dendritic cells might represent a niche harboring tolerant bacteria.

In a complementary approach, we have analyzed the cell types harboring viable *S.* Tm by fluorescence activated cell scanning (FACS). As bacterial loads in the cLN of ciprofloxacin treated mice were very low compared to the numbers of host cells (10^2^–2×10^3^ cfu versus 10^7^ cLN cells; Figure 1, Figure S4), the background noise of standard FACS was too high for a reliable analysis. Therefore, we have developed a FACS sorting protocol with subsequent plating of the sorted pools (Materials and Methods). This allowed highly sensitive detection of viable bacterial cells within subpopulations of cLN cells. For the experiment, we used CX_3_CR1^gfp/+^ transgenic mice that express *gfp* in particular monocyte populations [27]. These animals were infected for 3 d with *S.* Tm and treated (or not) during the last day with ciprofloxacin (2×62 mg/kg by gavage). Our data above indicated that about 1,000 *S.* Tm cells survive the antibiotic treatment in the cLN. The cLN was harvested, dissociated, and stained as described in Materials and Methods. Gating for CD45^+^CD3^−^CD19^−^ cells allowed us to focus on two particular cLN cell populations, the CD103^−^CX_3_CR1^+^ interstitial dendritic cells (iDCs) and the “classical” CD103^+^CX_3_CR1^−^CD11c^+^ dendritic cells (cDCs), which transport antigens from the mucosa to the cLN and are thought to be key antigen-presenting cells of the gut-associated immune system (Figure 3B; [28],[29]). Ciprofloxacin treatment affected the frequency of iDCs and cDCs in the cLN by no more than 2-fold (Figure 3C, left panel). The same was true for the *S.* Tm loads of the cDC (Figure 3C, right panel). In contrast, the *S.* Tm loads in the iDC plummeted by >10-fold in the ciprofloxacin-treated mice. These data are consistent with the microscopic analysis and indicated that cDC represent a key reservoir for tolerant *S.* Tm in the cLN of antibiotic-treated mice. The *S.* Tm population localized in cDC seems to feature a particularly high fraction of tolerant bacteria.

In order to assess the importance of the dendritic cells as a niche for tolerant *S.* Tm by independent means, we manipulated the number of dendritic cells via two well-established experimental systems. In the first experiment, dendritic cell numbers were reduced. For this purpose, we used a transgenic mouse (CD11c-DTR^+/−^; [30]) that allows dendritic cell depletion via diphtheria toxin (DTX; Materials and Methods). These mice (or nontransgenic controls) were infected with *S.* Tm, treated for 2 d with ciprofloxacin, and dendritic cells were depleted during the last day of the therapy. This reduced the load of *S.* Tm surviving ciprofloxacin treatment in the cLN (*p*<0.05; Figure 3D, left side). Conversely, increasing dendritic cell numbers by treating with Flt-3 ligand, a well-characterized agent expanding the dendritic cell population (i.e., cDC [31]), resulted in elevated *S.* Tm loads in the cLNs of ciprofloxacin-treated mice (Figure 3D, right side). These data confirmed dendritic cells as an important niche for antibiotic-tolerant bacteria in the cLN.

It had remained unclear how tolerant *S.* Tm cells do emerge. Is there a preexisting tolerant fraction of *S.* Tm cells that is enriched in the host's dendritic cells? Alternatively, the intracellular environment within a host cell might induce slow growth and tolerance in a fraction of the phagocytosed bacteria. In a first approach to address this question, we have performed in vitro infection experiments in bone-marrow–derived dendritic cells (BMDCs) and analyzed the fraction of *S.* Tm cells surviving ciprofloxacin treatment. We have compared the in vitro killing kinetics of the internalized bacteria with those of the inoculum. Bacteria residing within BMDCs displayed a higher fraction of tolerant cells than the bacteria of the inoculum (∼3% versus ∼0.06%; Figure S11). This may suggest that physiological changes during phagocytosis and/or phagosome maturation may induce tolerance. Alternatively, the BMDC may “enrich” tolerant bacteria from the inoculum—for example, by killing fast-growing intracellular *S.* Tm populations or by cell death of those BMDCs phagocytosing fast-growing bacteria. Anyway, our observations are in line with earlier work detecting slow-growing *S.* Tm cells during ex vivo infection of macrophages and BMDCs [32],[33]–[36]. However, it had remained unclear if this would also occur in vivo.

We hypothesized that a slow in vivo growth rate may explain the pathogen's antibiotic tolerance in the cLN. This seemed plausible as earlier work had shown that many bacterial species can become antibiotic tolerant in the stationary phase when bacterial growth is extremely slow [1]–[7],[18],[37],[38]. Moreover, tolerant *Salmonella* populations were indeed detectable in fluoroquinolone-treated mice ([17],[39]; this work). Though suggestive, none of these studies was able to establish that tolerant pathogen populations in an infected host were indeed growing slowly. Therefore, we needed evidence linking pathogen growth rates in vivo to the observed recalcitrance to antibiotic treatment.

Determining the replication rate of bacteria in vivo constitutes a challenge. Simply counting the number of bacteria in an anatomical compartment does not allow disentangling replication, migration, and clearance [40]. However, parameters characterizing these processes can be estimated with a population dynamical analysis of infection experiments conducted with mixtures of differentially tagged strains. In theory, fluctuations in the proportions of the differentially tagged strains are indicative of population bottlenecks during migration between and replication within host compartments, and can be used to estimate the population dynamical parameters (Materials and Methods).

**Figure 4:**
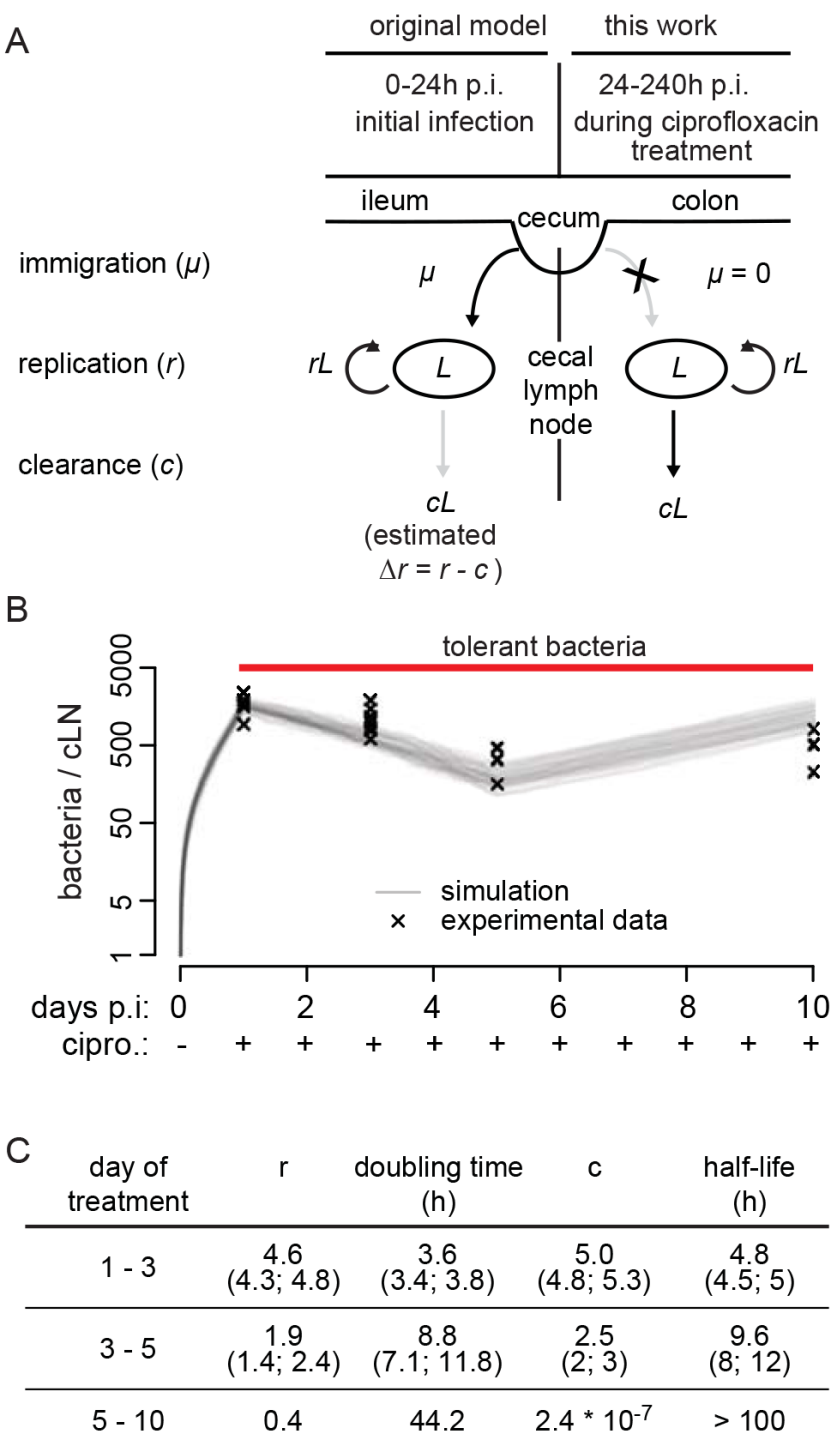
*S.* Tm^WITS^ infection and a modeling reveal slow growth of tolerant bacteria. (A) Schematic representation of the stochastic birth-death process modified by immigration and its parameterization (for details, see Materials and Methods). During the first 24 h p.i., we analyzed cLN colonization dynamics in absence of ciprofloxacin. Ciprofloxacin treatment was started by 24 h p.i. and continued until day 3, 5, or 10, as indicated. The model for analyzing the latter data is displayed on the right side. (B) Mice were infected with *S.* Tm^WITS^ and treated with ciprofloxacin (2×62 mg/kg/d by gavage) from day 1 p.i. until the indicated end of the experiment (day 1, *n* = 49; day 3, *n* = 28; day 5, *n* = 28; day 10, *n* = 28 data points). CFU determination and analysis of tag abundance using rtqPCR (experimental data, Table S2) was used to fit the mathematical model. Graphic display of experimental data (x) and 100 simulations (grey lines). (C) Parameters (with confidence intervals) derived from fitting the mathematical model to our experimental data. *r*, replication rate; doubling time, ln2/*r*×h; *c*, clearance rate; half-life, ln0.5/*c*×h.

To that end, we devised a novel approach extending our recent work on the population dynamics of cLN colonization by *S.* Tm in the mouse model for complicated Salmonellosis [40]. The original method employed a defined mixture of differentially tagged, isogenic *S.* Tm strains—that is, wild-type *S.* Tm (SL1344 *S.* Tm) spiked with limiting amounts of phenotypically identical strains (*S.* Tm^WITS^). These strains are isogenic to wild-type *S.* Tm, except for a 40-nucleotide sequence, which serves as an identifiable neutral marker that can be quantified by real-time qPCR [40],[41]. The inoculum contained a total of seven different *S.* Tm^WITS^, each carrying a unique 40 nucleotide sequence (mixed in a 1∶1∶1∶1∶1∶1∶1 ratio) and a 20-fold excess of untagged wild-type *S.* Tm ([40], this work; Figure S12). In our initial work, this approach was used to estimate the net bacterial replication rate in the cLN by analyzing the *S.* Tm^WITS^ infection data with a stochastic birth-death model extended by immigration [40]. The model predicted the fraction of strains that successfully migrated to the cLN and their population size as a function of the rate of immigration, *μ*, replication, *r*, and clearance, *c* (Figure 4A). Fitting this model to our experimentally determined number of each tagged strain in the cLN, we could infer rates at which bacteria immigrate into the cLN (298 bacteria during the first 24 h p.i.) and replicate therein. The validity and robustness of this approach was verified analyzing *S.* Tm^WITS^ infections in wild-type and knockout mice [40]. In our present analysis of the tolerant *S.* Tm cells in the cLN, we used these parameters to estimate the composition of the *S.* Tm population at the beginning of the ciprofloxacin treatment (Figure 4A,B), and extended this approach to quantify the growth rate of the *S.* Tm cells surviving the ciprofloxacin treatment in the cLN (Figure 4A, Figure S12, Materials and Methods, Text S1).

The population dynamics experiment had two phases. First, infection was established for 24 h with a mixture of *S.* Tm^WITS^ (*n* = 35–49 data points per group; 7 differentially tagged *S.* Tm^WITS^ strains). Second, high-dose ciprofloxacin treatment was started at 24 h p.i. This rapidly diminished pathogen loads in the gut lumen (Figure S2A), and thus, de novo immigration into the cLN can be neglected in our analysis of this second phase of the experiment. The number of each *S.* Tm^WITS^ in the cLN was determined experimentally at days 1, 3, 5, and 10 postinfection (Table S2). From these data, we could determine both the doubling time and the clearance rate of the surviving bacteria in the cLN for each time period separately (i.e., days 1–3, 3–5, and 5–10; Figure S12). During the first period of the treatment (days 1–3), susceptible bacteria were killed and the persistent bacteria remained in the lymph node. Thus, the population dynamics will represent the aggregate of both processes (that is, the average over the dynamics of the two subpopulations, not the aggregate of replication and clearance), and the apparent in vivo doubling time was about 3.6 h. In the subsequent two periods, we could analyze the population dynamics of the tolerant *S.* Tm cells in isolation. Indeed, the doubling time increased to 8.8 h (days 3–5) and 44 h between days 5 and 10 of the therapy (Figure 4B,C). In this period, the clearance rate dropped to extremely low levels (<0.01/h), indicating that the half-life of bacteria that survived the ciprofloxacin in the cLN exceeded 100 h. We do not know if *S.* Tm forms different types of tolerant subpopulations. This might explain why the doubling time and the half-life of the tolerant *S.* Tm cells increased between days 3 and 10 of the treatment. In any case, these data established that the antibiotic-tolerant bacteria enter a persistent state characterized by extremely slow replication and virtually no clearance.

**Figure 5:**
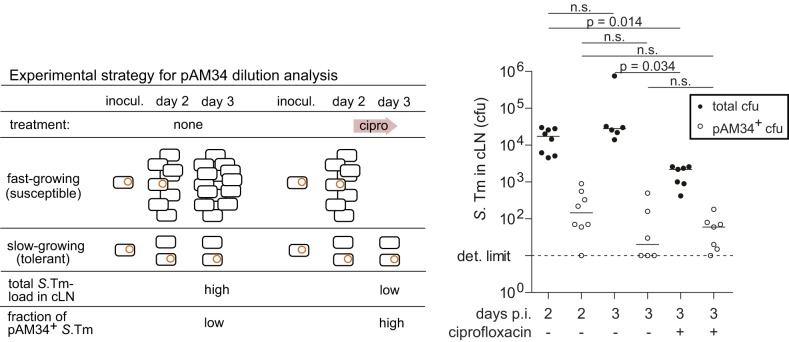
Plasmid dilution experiment verifying the slow growth rate of tolerant *S.* Tm cells in vivo. (Left) Experimental strategy. (Right) Experimental data. Three groups of C57BL/6 mice were infected for 2 d or 3 d with *S.* Tm(pAM34), as indicated. The third group was treated with ciprofloxacin during the third day (2×62 mg/kg/d by gavage). Total *S.* Tm loads (closed symbols) and *S.* Tm(pAM34) (open symbols) in the cLN were determined by plating. Dashed line, detection limit. *p*, nonparametric statistical analysis. n.s., not significant.

To confirm the slow growth rates of tolerant *S.* Tm cells in vivo, we employed a plasmid-dilution strategy (Figure 5, left side). pAM34 is an IPTG-addicted plasmid that stops replication as soon as IPTG is removed from the environment, including all mouse tissues analyzed (pAM34, Amp^R^, [42]). This type of plasmid can be used to verify the slow growth rate of the ciprofloxacin-tolerant *S.* Tm subpopulation. Such tolerant cells should survive the antibiotic and should feature a high fraction of cells retaining pAM34.

Three groups of mice were infected with *S.* Tm (pAM34). One group was sacrificed at day 2 p.i. to assess cLN loads and the fraction of bacteria still retaining the plasmid. Here we observed the typical pathogen loads of approximately 10,000 cfu in the cLN (Figure 5; compare to Figure S4). Approximately 100 of these bacteria were still carrying pAM34 (Figure 5, open symbols). The second group was left untreated, analyzed at day 3 p.i., and served as a control. The third group was treated with ciprofloxacin from day 2 on and analyzed at day 3 p.i. Here, we observed significantly reduced total pathogen loads in the cLN (1,000–2,000 cfu; *p*<0.05), while the total loads of pAM34 carrying *S.* Tm cells did not differ significantly from those at day 2 or the control mice at day 3 p.i. (50–100 bacteria; *p*≥0.05). These findings are consistent with our data presented above and indicate that *S.* Tm does form a susceptible and a tolerant subpopulation in the cLN. Based on that data, we reasoned that approximately 80%–90% of the total cLN bacteria displayed the fast-growing/susceptible phenotype, while 10%–20% belonged to the slow-growing/tolerant phenotype. If slow-growing bacteria would exist in the cLN even in the absence of ciprofloxacin, most of the pAM34 should reside in such slow-growing/tolerant *S.* Tm cells at day 2 p.i., before the onset of ciprofloxacin treatment. These should survive the ciprofloxacin treatment and thereby account for the high loads of pAM34-positive *S.* Tm at day 3 p.i. This is supported by the data from the group of mice infected for 2 d with *S.* Tm (pAM34) and treated with ciprofloxacin during the third day of the infection (Figure 5). Here the susceptible *S.* Tm cells were eliminated, while tolerant cells prevailed and the number of bacteria retaining pAM34 did not drop significantly. This confirmed that the ciprofloxacin-tolerant cells were harboring the bulk of the pAM34 plasmid observed at day 2 p.i. and provided in vivo evidence that the slow-growing/tolerant subpopulation exists within the cLN (presumably within classical dendritic cells) even in the absence of ciprofloxacin. These findings are in line with the ex vivo data by Helaine and Holden, who observed by elegant fluorescence microscopy techniques that some fraction of internalized *S.* Typhimurium cells grow at a strikingly low rate [32].

From the pAM34 data of the ciprofloxacin-treated mice we could roughly estimate the doubling time of the tolerant bacteria. We assumed that the bacteria had reached the cLN by 12 h p.i., resided at this site for 60 h, and displayed the tolerant, slow-growing phenotype throughout these 60 h. On this basis we could take the pAM34 frequencies of the inoculum and of the cLN resident bacteria at day 3 p.i. to estimate a doubling time of 8.9 h for the tolerant phenotype. This value is strikingly similar to that observed in our population dynamics experiments [8.8 h (7.1–11.8 h) at days 3–5; Figure 4C] and further supports that tolerance is growing indeed quite slowly in vivo.

A second phenomenon observed in the ciprofloxacin treatment was a reduced pro-inflammatory gene expression signature of the cLN (reduced *cxcl2*, *ifng*, *s100a9*, *lcn2* expression; Figure S13). Overall, these data suggested that the antibiotic treatment may have two effects in parallel. First of all, it reduces the total bacterial burden in the cLN by killing the susceptible bacteria. This dampens the pro-inflammatory response in the tissue. Second, it might be of importance that the tolerant bacteria do not just survive the ciprofloxacin treatment. They may in fact face a reduced pro-inflammatory defense in the cLN (Figure S13). One might speculate that this further enhances the survival of tolerant *S.* Tm cells in the tissue. It will be an important task for future work to dissect which environmental signals do induce slow growth/tolerance by *S.* Tm and whether/how this is affected by pro-inflammatory signaling in the infected tissue.

**Figure 6:**
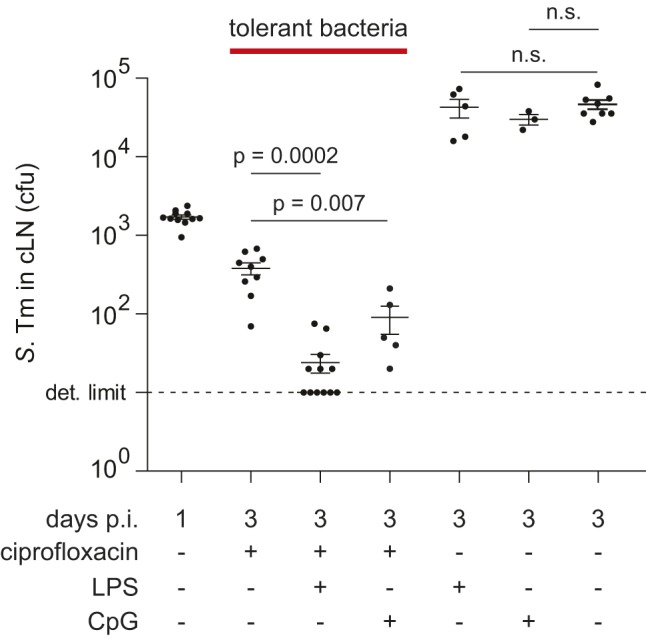
LPS or CpG treatment reduces the number of ciprofloxacin-tolerant bacteria in the cLN. C57BL/6 mice were infected for 1 d or 3 d with wt *S.* Tm (5×10^7^ cfu by gavage). Ciprofloxacin treatment (2×62 mg/kg/d) was started at day 1 p.i. and continued until the end of the experiment, as indicated. The indicated groups were treated with a single dose of LPS (5 µg intraperitoneally) or CpG (100 µg intraperitoneally) at day 2 p.i. Cfus in the cLN were analyzed by plating. For gut content and spleen loads, see Figure S14. Each symbol represents one mouse. Dashed line, detection limit. *p*, nonparametric statistical analysis (Mann–Whitney U test). n.s., not significant.

Finally, we were interested to identify a strategy for reducing loads of ciprofloxacin-tolerant pathogens in wild-type hosts. Earlier work had indicated that antibiotics and the host's immune system may cooperate in eliminating pathogens during antibiotic therapy [43],[44]. Furthermore, the antibiotic-treated lymph node displays a signature of reduced innate immune defense (Figure S13). Based on such observations, it has been speculated that triggering innate responses during the antibiotic treatment may allow reducing the number of persistent bacteria [1]. However, to the best of our knowledge, this has not been substantiated by experimental data in vivo. To test this hypothesis, we applied a PRR ligand. Mice were infected for 1 d with *S.* Tm and treated with ciprofloxacin alone (2×62 mg/kg per day), LPS alone (one dose of 5 µg at 48 h p.i.), or ciprofloxacin and LPS. Indeed, a single dose of LPS applied during the ciprofloxacin treatment elicited within 2 h an innate immune response in the cLN and significantly reduced the number of tolerant bacteria (Figure 6, Figure S13, Figure S14). In six animals the pathogen loads were reduced below the limit of detection (red arrowhead; Figure 6). Loads of tolerant bacteria were also reduced in the cecum wall and the cecal patch (Figure S15). However, it had remained unclear whether elimination of tolerant bacteria was attributable to direct or to indirect (paracrine) signaling to the infected host cell. In a first approach, we have generated mixed bone marrow chimeric mice. These mice displayed 50%–70% *tlr4* ^−/−^ cDC and 30%–50% wt cDC. Only the latter should directly respond to LPS, while both should respond to paracrine signaling. When these mice (or wt control animals) were infected with *S.* Tm(pAM34) and treated with ciprofloxacin and LPS, we observed the same strong reduction of the cLN loads (and pAM34 retention), as in wild-type mice (Figure S16). This suggested that LPS can affect tolerant *S.* Tm cells via an indirect, paracrine mechanism.

A second PRR-ligand, CpG, was also capable of reducing the tissue load of tolerant *S.* Tm cells (Figure 6). These data demonstrated that the persistent bacteria lodged within the cLN dendritic cells are indeed susceptible to innate immune responses and that the PRR-agonists LPS and CpG can supplement antibiotic treatment.

## Discussion

In conclusion, high-dosed ciprofloxacin is capable of reducing *S.* Tm populations from most sites of the infected host. However, a fraction of the bacteria survives the treatment. In the cLN, approximately 10% survive in a persistent state and the “classical” cDCs seem to represent a niche harboring a very high fraction of ciprofloxacin-tolerant *S.* Tm cells.

It is interesting to note that the cDCs play an important role in antigen sampling by the gut-associated immune system. Based on these observations, it is tempting to speculate that it is exactly the dendritic cells' function in adaptive mucosal immunity—that is, microbe transport to the draining lymph nodes and prolonged antigen presentation [25],[45]–[47], which might generate (e.g., induce or select for) the tolerant *S.* Tm subpopulation. It remains to be shown whether this also holds true for human patients treated for complicated infections by *Salmonella* spp. or other gut pathogens. Such pathogen–host interactions and their effects on antibiotic therapy will be of broad importance for infection biology and represent an interesting topic for future work. The experimental framework developed using the infection model for complicated Salmonellosis could enable such studies. Considering the broad variety of pathogens, the diversity of niches within the host, the multitude of virulence factors, and the vastly differing average growth rates within hosts displayed by pathogens, it seems likely that such work will reveal pathogen-specific adaptations and common mechanisms explaining recalcitrance to antibiotic therapy. Clearly, deciphering the population dynamics of pathogens within their hosts will be of key importance for understanding the infection process and developing improved therapeutic strategies.

It is still not entirely clear how bacteria can obtain an antibiotic-tolerant phenotype, whether different bacteria might employ different strategies, and how host–cell interactions might affect tolerance. Originally, a tolerant subpopulation was discovered in *S. aureus* [48]. Later, this phenotype has been observed in most bacterial species analyzed [3],[49]. Bacterial populations of identical genotypes do generally feature a fraction of cells that are more resistant to a given antibiotic than the others. The size of this fraction can increase in response to external stimuli, including nutrient limitation, quorum sensing signals, antibiotics, or oxidative stress. This engages signaling mechanisms within the bacterial cell, including the SOS response and toxin–antitoxin systems, which are finally thought to reduce the bacterial growth rate and/or elicit tolerance [3]. In conjunction with earlier observations [20],[32]–[36], our data suggest that slow growth and concomitant antibiotic tolerance of *S.* Typhimurium in vivo are attributable to the peculiar interaction with dendritic cells. The high prevalence of tolerant *S.* Tm cells in cDC might result from the combination of long-term survival of *S.* Tm in this cell type (but not in other monocytic phagocytes and granulocytes; [47],[50]) and the slow growth of the pathogen within these cells in vivo. Formally, we cannot exclude that cDC might also “select” for slow-growing, tolerant *S.* Tm cells. Anyhow, upon ciprofloxacin exposure, this bacterial subpopulation survives and might even benefit from the antibiotic-inflicted reduction of the overall tissue burden. Future work will have to identify the host cellular features and the bacterial responses establishing tolerance. This will be of importance as such cells can cause relapses after the end of an antibiotic therapy.

The presence of ciprofloxacin-tolerant cells in gut mucosa, the cecal patch, the cLN, and the spleen may suggest that relapses can be “seeded” from various reservoirs. The site of reseeding may affect the relapsing disease progression. This will be a challenging topic for future work, as site specificity of the “seeding” step is difficult to establish, at least by end point assays as used in our current study. It is interesting to note that cell transfer into the peritoneal cavity of the recipient mouse led to seeding of the spleen and the liver (Figure 2). In contrast, no (or few) bacteria were detected in the cLN or the gut lumen of the recipient mice within 4 d. This is consistent with the route of drainage of the peritoneal cavity, which should initially seed the spleen and the liver, while gut lumen and the gut draining mesenteric lymph nodes would represent sites of subsequent dissemination at later stages of the relapse.

Finally, our data establish that persistent bacteria are not invulnerable. Triggering of innate immune responses can decimate the loads of persistent *S.* Tm in the cLNs of antibiotic-treated mice. The TLR4 mixed bone marrow chimeras indicated that this may occur via indirect, paracrine signals. Clearly, more work is required to establish the underlying cellular mechanisms. Furthermore, as Toll-like receptor agonists may evoke significant unwanted side effects, alternative strategies for inducing innate immune responses should be explored. CpG-based drugs are presently tested in clinical trials for cancer therapy [51]. This may foster future work optimizing the augmentation of antibiotic treatment by PRR ligands and may lead to novel antibacterial therapies with increased in vivo efficacy.

## Materials and Methods

### Bacterial Strains and Growth Conditions

The wild-type strain SB300 used in this study is an SL1344 derivative and has been described previously [52]. This strain was resequenced and found to be 100% identical to the published wt SL1344 sequence (GenBank accession FQ312003.1) except for two nucleotide changes at positions 635606 (T→A, Glu→Val in a putative mannose-specific PTS system enzyme IIAB) and 1756092 (G→A, Asp→Asn in *yciT*, a putative regulatory protein), respectively [53]. *S.* Tm strain ATCC14028 has been described [54],[55].

Wild-type isogenic-tagged strains (*S.* Tm^WITS^, SB300 background [41]) and the RFP expression plasmid pDsRed (pACYC184 backbone, *lac* promotor) have been described [23]. For infection experiments, the bacteria were grown overnight in LB broth (0.3 M NaCl), subcultured for 4 h, and suspended in cold PBS as described previously [56].

### MIC Testing for Ciprofloxacin

A stationary culture (37°C, aerated, in LB) was diluted 1∶100,000 and 10 µl were spotted on an LB agar plate containing ciprofloxacin. The first concentration to inhibit visible growth was considered to be the MIC, as described in [57]. The MIC was determined to be <0.03 µg/ml, comparable to earlier results [17].

### Mouse Experiments

B6-Tg(Itgax-EYFP) mice expressing EYFP under the CD11c promoter (CD11c-YFP; [26]), CD11c-DTRtg heterozygous transgenic mice (B6.FVB-Tg(Itgax-DTR/EGFP)57Lan/J; CD11c-DTR^tg^; [30]), CX_3_CR1^gfp/+^ heterozygous transgenic mice (CX_3_CR_1_^tm1Litt^) [27], TLR4^−/−^ (TLR4^lps-del^) mice [58], and wild-type C57BL/6 mice were bred and kept specified pathogen free in individually ventilated cages (RCHCI, ETH Zürich). Ciprofloxacin (ciproxine 500, Bayer Schering Pharma) was dissolved in water, sterile-filtered, and the concentration was quantified by UV spectrometry (A_271 nm_ = 30,614 l×mol^−1^×cm^−1^; [59]). It was applied intragastrically twice daily, as indicated (2×62 mg/kg per day). LPS from *S.* Typhimurium (Sigma L2262-5MG; 5 µg per mouse) was dissolved in PBS and injected intraperitoneally 24 h after the beginning of ciprofloxacin treatment. All animal experiments were approved (licences 201/2007, 223/2010, Kantonales Veterinäramt Zürich) and performed as legally required. Streptomycin-pretreated mice (20 mg/animal) were infected by gavage (5×10^7^ CFU; [16],[23]). Live bacterial loads (colony forming units, cfus) in cecum draining lymph node (cLN), spleen, liver, and cecal content were determined by plating [60]. The small intestine draining lymph node has not been analyzed, as it is only occasionally colonized by a small number of bacteria during the first 1–2 d of infection. Minimal detectable levels were 10 cfu/cLN, 10 cfu/spleen, and 100 cfu/g stool/cecal content. CD11c-DTR^tg^ heterozygous transgenic mice and nontransgenic littermate controls were treated with DTX (Sigma-Aldrich, dissolved in PBS, 100 ng/25 g body weight; i.p. injection) 24 h before termination of the experiment. The depletion efficiency has previously been shown to be about 80% in the intestinal lamina propria and more than 90% in spleen and mesenteric lymph nodes [23]. It was verified by FACS analysis of cLNs. To expand dendritic cell numbers, mice were treated for 3 subsequent days with 10 µg of hFLT3-L (kindly provided by Christian Münz or from eBioscience), starting 2 d prior to infection. Expansion of the DC population was verified by FACS of peripheral blood 24 h after the last injection (see Materials and Methods, FACS).

### Fluorescence Microscopy

Cecum lymph node tissue samples from CD11c-YFP mice infected with *S.* Tm pDsRed were incubated for 1 h in 4% paraformaldehyde dissolved in PBS at 4°C, equilibrated 4 h in 20% sucrose dissolved in PBS at 4°C, and snap-frozen in O.C.T. compound (Sakura). The 20 µm cryosections were air-dried, blocked (10% goat serum, PBS), stained with DAPI (Sigma-Aldrich), and mounted (Vectashield; Vector Laboratories). Images from immunofluorescence stainings were recorded with a Zeiss Axiovert 200 microscope, an Ultraview confocal head (PerkinElmer), and a krypton argon laser (643-RYB-A01, Melles Griot, Didam, The Netherlands). Infrared, red, and green fluorescence was recorded confocally, and blue fluorescence by epifluorescence microscopy. Images were transformed to the colors indicated, superimposed, and analyzed with Volocity 5.0.3. (Improvision, Coventry, UK).

### Statistical Analysis

Statistical analysis was performed using the exact Mann–Whitney U Test (Prism Version 5.04). *p*<0.05 (two-tailed) was considered as statistically significant as described [16]. The exact Fisher's test (R 2.15.1) was used to determine significance of reinfection/relapse efficiency in Figure 2. For analysis of the WITS experiment, please refer to the dedicated Materials and Methods section.

### Wild-Type Isogenic Tagged Strain Quantification

Mice were infected according to our standard infection protocol [16] with a uniform mixture of 7 *S.* Tm^WITS^ [41], diluted with a 20- or 100-fold excess of untagged *S.* Tm. Mice were killed by cervical dislocation and the cLN was aseptically removed and homogenized in 500 µl of ice-cold PBS (0.5% Tergitol, 0.5% bovine serum albumin) by using a Potter homogenizer. We inoculated 250 µl of the cLN homogenate into an LB overnight culture containing 50 µg/ml Kanamycin to enrich for *S.* Tm^WITS^, and 150 µl were plated on MacConkey agar plates to determine the cfu. Chromosomal DNA from enrichment cultures was isolated using QIAamp DNA Mini Kit (Qiagen, Cat. No. 51306) and subjected to rtqPCR using FastStart Universal SYBR Green Master (Rox) (Roche, 13206900) with primers and temperature profiles as described [41]. The ratio of present WITS was multiplied with the number of cfus recovered to determine the absolute number of each tagged strain in the cLN.

### Quantifying Generations Using the Nonreplicative Plasmid pAM34

*S.* Tm (pAM34) was cultured for 5 h in the presence of IPTG (1 mM), subcultured twice 1∶20 into LB w/o IPTG for 3 h, spun down (10,000 rpm, 4°C, 5 min), resuspended in ice-cold PBS, and inoculated orally into mice (5×10^7^ cfu). In order to estimate the number of pAM34 plasmids per bacterial cell of the inoculum, serial 1∶100 dilutions of the inoculum were inoculated into fresh LB w/o IPTG, grown to stationary phase, and plated onto MacConkey plates containing streptomycin or ampicillin and IPTG. The observed percentage of ampicillin-resistant colonies obtained from each differentially inoculated culture was used to generate a standard curve linking pAM34 loss to the numbers of divisions of a given *S.* Tm population that had grown out from the same inoculum.

Mice were infected and treated with antibiotics as described under mouse experiments. At the end of the experiment, we aseptically removed the cLN and determined the total loads of viable *S.* Tm by plating on MacConkey agar (50 µg/ml streptomycin) and the loads of *S.* Tm(pAM34) by plating on MacConkey agar (100 µg/ml ampicillin, 1 mM IPTG). For estimating the growth rate of the ciprofloxacin-tolerant *S.* Tm cells in vivo (Figure 5), we used the standard curve described above for estimating the numbers of generations that these bacteria had undergone during the infection process.

### BMDCs

Femur and tibia of C57BL/6 mice were excised, cleaned, and flushed with RPMI 1640 (10% FCS, 50 µM β-mercaptoethanol, 50 µg/ml streptomycin). Cell suspension was incubated for 30 min (37°C, 5% CO_2_), supernatant was removed, spiked with GM-CSF (200 U/ml), and seeded into 24-well plates. Medium containing GM-CSF was replaced after 2 d. On day 3, all nonadherent and loosely adherent cells were removed, and medium was replaced. BMDCs were used after 5 d in culture.

### In Vitro Killing Curves

BMDCs were infected for 20 min with *S.* Tm (4 h subculture of 12 h overnight culture in 0.3 M NaCl LB), washed, and incubated for 13 h under gentamycin. We added 10 µg/ml ciprofloxacin, incubated as indicated at 37°C, 5% CO_2_. Similarly, an OD = 0.2 subculture of *S.* Tm was treated with 10 µg/ml ciprofloxacin, incubated at 37°C, and surviving bacterial cfus were determined each hour by plating.

### FACS

Single-cell suspensions of cLNs were generated by incubating the tissue in DMEM (50 µg/ml Liberase and 20 µg/ml Dnase) for 30 min at 37°C and subsequently run through a 100 µm cell strainer. For sorting, samples were stained with CD90.2-PercP (BioLegend, 105322), CD11b-APC-Cy7 (BioLegend 101220), and B220-APC (Becton Dickinson 553093). We sorted 2×10^6^ lymph node cells into CD90.2^−^CD11c^−^ or CD90.2^−^CD11c^+^ using a FACSAria cell sorter (Becton Dickinson), washed them, and lysed them, and bacterial loads were determined by plating. To determine the efficiency of Flt3-L mediated expansion of DCs (CD4^−^CD8^−^B220^+^CD11c^+^: PBS, 0.3%; hFLT3-L, 0.6%), blood samples were stained with B220-APC (Becton Dickinson 553092), CD11c-APC-Cy7 (BioLegend 117324), CD8α-PacB (BioLegend 100725), and CD4-PE (Becton Dickinson 553049). To asses efficiency of DC depletion by DTX injection (CD45.2^+^CD4^−^CD8^−^B220^−^CD11c^+^: wt mice, 5.3%; DTR^+^ mice, 2.6%), cLNs were stained with CD45.2-APC (BioLegend 109813), CD11b-APC-Cy7 (BioLegend 101226), CD11c-FITC (Becton Dickinson 557400), CD4-PercP (Becton Dickinson 553052), CD8-PercP (Becton Dickinson 553036), and B220-PercP (Becton Dickinson 553093) and FACS analyzed. The following antibodies of Biolegend were used for cell surface staining to sort myeloid subsets for Figure 3: CD45 (30-F11), CD3 (17A2), CD19 (6D5), CD103 (2E7), CD11c (N418), and Gr-1 (RB6-8C5). Live cells were determined as SYTOX− cells (Invitrogen). At least 10×106 lymph node cells were analyzed and 104 to 105 cells sorted into (1) CD45+ CD3− CD19− CD103− CX3CR1+ myeloids, (2) CD45+ CD3− CD19− CX3CR1− CD103+ CD11c+ Gr1− conventional DCs, and (3) CD45+ CD3− CD19− CD103− CX3CR1− CD11c− Gr-1+ myeloids using a BD FACSAria III cell sorter. Sorted lymph noded cells were pelleted, washed, lysed, and bacterial cfus were determined by selective plating.

### Real-Time Quantitative PCR

The excised cLN was placed in 600 µl RNAlater (Qiagen) and shock frozen at −80°C. Total RNA extraction was done using the RNeasy mini kit (Qiagen) with RNase-free DNase digest (Qiagen). For reverse-transcription of 1 µg mRNA aliquots, the RT^2^ HT First Strand cDNA Kit (Qiagen) was used. Custom RT^2^ Profiler PCR Arrays (Qiagen) were run with RT^2^ SYBR Green ROX FAST (QIAGEN) on an Applied Biosystems 7900HT Fast Real-Time PCR System to amplify the resulting cDNA. Relative mRNA levels (2-ΔCq) were determined by comparing the PCR quantification cycle (Cq, determined with the Software SDS 2.2.1) for genes related to inflammation and defense against *S.* Typhimurium infection (the selection is based on [61]) with the reference gene Actb. The differences in their Cq cycles were calculated (ΔCq). In all experiments, the upper limit of Cq was fixed to 35 cycles. Then, the fold actin was calculated and plotted. Each sample was controlled for mouse genomic DNA contamination. All DNA-positive data were excluded from further analysis.

### Mathematical Modeling and Statistical Analysis

The inoculum in our infection experiments contained a minority population consisting of seven distinct WITS. Measuring the population sizes of each WITS in the cLN reveals the stochasticity that is inherent in the colonization dynamics but is “averaged out” in measurements of the majority population. By revealing the stochasticity, WITS allow us to disentangle dynamical parameters that could not be estimated individually. In a previous study [40] we showed that a minority population of WITS in the inoculum allows us to disentangle immigration and net replication rates. Here, we can exploit the stochasticity to separate replication and clearance rates in the cLN during antibiotic treatment.

There are many potential ways of using the complex experimental data we generated for mathematical modeling. We chose to use only the WITS data to estimate the rates of replication and clearance. We also have measurements of the total bacterial population size in the cLN, which were determined independently from the population sizes of the WITS. Although we could have used the data on the total bacterial population size to further refine our parameter estimates, we decided to use them to validate our model fit instead (see Figure 4B). The consistency between experimental data and model prediction corroborates our parameter estimates for replication and clearance rates under antibiotic treatment.

Mathematically, we describe the colonization of the cLN by *S.* Tm by a stochastic birth-death-immigration model ([62] see also [40]). Let *L* be a random number that denotes the number of a unique WITS in the cLN. In the model, WITS are assumed to migrate from the cecum to the draining lymph node at a rate *μ*, replicate in the lymph node at a rate *rL*, and be cleared at a rate *cL*. All these processes are assumed to be stochastic. The transition probabilities of our model are then given by the following equations:
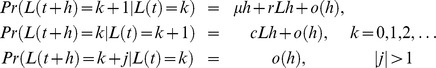
A diagram showing the processes incorporated into our model is shown in Figure 4A.

In [40], we derived a likelihood function and used it to estimate parameters of our model for cLN colonization within the first day after intragastric infection. We estimated that during the first day, 298 bacterial cells enter the cLN, and that the bacterial population grows at a rate of 2.82 per day (corresponding to a population doubling time of 5.9 h; no ciprofloxacin applied during this period).

To estimate replication and clearance parameters during antibiotic treatment, we combined the above estimates (characterizing the early colonization dynamics) with new experiments. In these experiments, mice were infected with mixed inocula containing seven uniquely tagged *S.* Tm^WITS^. Treatment was started 1 d after infection and maintained until day 3, 5, or 10. Because treatment kills almost all bacteria in the gut, we set *μ* = 0—that is, we assume that, once treatment has started, the influx of bacteria into the cLN is negligible. As a useful side effect of having only two parameters to estimate, we do not face any problem of identifiability as for the early colonization parameters [40]. As treatment starts 1 d after inoculation, the cLN is not empty but contains approximately 1,000 bacterial cells. To derive a likelihood function, we therefore need to calculate the probability generating function for arbitrary distributions of initial bacterial population sizes. (In contrast, for the derivation of the likelihood function for early colonization, we could assume an empty cLN.) For the general initial condition *f*(*s*, *t* = 0) = *g*(*s*) that describes an arbitrary distribution of initial bacterial population sizes, the probability generating function is:
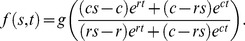
(1)We first estimated replication and clearance rates from the WITS data sampled at day 3 after infection. We denoted these rates as *r*_3_ and *c*_3_, respectively. We assume that the number of WITS in the cLN at day 1 after infection is distributed according to the probability distribution:
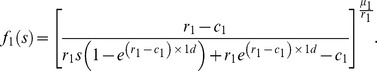



Hereby, the parameters *μ*_1_, *r*_1_, and *c*_1_ characterize the dynamics from day 0 to day 1 after inoculation without antibiotic treatment. This assumption leads to better estimates than assuming a fixed bacterial population size in the cLN 1 d after inoculation.

From Equation (1) with *g*(*s*) = *f*_1_(*s*), we can determine the probability generating function under treatment. Unfortunately, the state probabilities *p_k_*, which are needed for likelihood-based inference of the model parameters, cannot be determined in closed form. This is in contrast to the scenario considered in our previous study [40], in which the process started with an empty cLN. We therefore calculated the state probabilities numerically using Fourier transformation of the characteristic function. The characteristic function can be obtained from a probability generating function by substituting *e^iθ^* for the dummy variable *s*. The state probabilities *p_k_* are related to the characteristic function as 
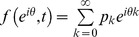
. We can invert this relation and calculate the state probabilities *p_k_* using the normalized discrete inverse Fourier transform (see also [63]):
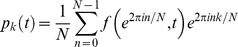



The parameter * N* should be a large number. The larger * N*_,_ the more accurate the state probabilities. In our implementation, we have chosen * N* = 2^14^. With these values, the likelihood and maximum likelihood estimates of the parameters reach a relative accuracy of 10^−8^. To obtain maximum likelihood estimates from the state probabilities *p_k_*, we followed the same procedure as in our previous study [40]. The resulting estimates for *r*_3_ and *c*_3_ constitute averages of the replication and clearance rate over the period from day 1–3.

We follow an analogous procedure to obtain parameter estimates for the average replication and clearance rates over the periods day 3–5 and day 5–10 from the experimental enumeration of the *S.* Tm^WITS^ at days 5 and 10, respectively. To that end, we set *g*(*s*) in Equation (1) to the probability generating function describing the state of the system at the end of the preceding period. These parameters are denoted as *r*_5_, *r*_10_, *c*_5_, and *c*_10_.

### Implementation

All the probability-generating functions and likelihoods described here were implemented in the R language of statistical computing [64]. To obtain the maximum likelihood estimates, we used the R-function optim(). Confidence intervals for the estimates were obtained with a bootstrap routine from 200 replicates. To simulate the process as shown in Figure 4B, we used the R-package GillespieSSA [65]. In these simulations, we used the parameter estimates of *r*_1_, *r*_3_, *r*_5_, *r*_10_, *c*_1_, *c*_3_, *c*_5_, and *c*_10_ that we had obtained from the data. An R-package containing the datasets, likelihood, and simulation functions is provided as Protocol S1 and described in Text S1. A systems biology markup language (SBML) version of the simulation model was also deposited in BioModels Database and assigned the identifier MODEL1312170001.

### Ciprofloxacin Pharmacokinetic Analysis

Mice were treated by gavage with 2.5 mg of unlabeled ciprofloxacin spiked with 75 µg of C-14 labeled ciprofloxacin (kindly provided by Bayer HealthCare). Mice were sacrificed 2, 8, and 12 h later, and the indicated tissues were dissolved in 1 ml Solvable (PerkinElmer 6NE9100; 55°C, overnight). We added 100 µl of 30%–35% H_2_O_2_ and incubated it for 1 h at 37°C. Samples were mixed with 15 ml Ultima Gold scintillation cocktail (PerkinElmer: 6013329), and C-14 was measured using a PerkinElmer Tri-Carb scintillation counter.

## Supporting Information

Figure S1 **Control experiment verifying that high-dose ciprofloxacin treatment achieves concentrations of >>MIC in vivo.** Pharmacokinetic analysis of the tissue concentration of ciprofloxacin. As described in Materials and Methods, we applied a single dose of ^14^C-labeled ciprofloxacin and analyzed the intensity of the ^14^C signal 2 h, 8 h, or 12 h later. This verified that the ciprofloxacin levels in the cLN were >50-fold higher than the MIC throughout the therapy.
(EPS)

Figure S2 **Kinetics of** ***S.*** **Tm elimination by ciprofloxacin.** (A) The 3 h killing curve of the stool *S.* Tm population after application of a single dose of ciprofloxacin (62 mg/kg p.o.). (B) Killing curve of *S.* Tm in the cLN after the onset of ciprofloxacin treatment. The *S.* Tm population shows fast initial killing (0–2 h) and much slower killing kinetics between 2 h and 3 h (persistence of 50–100 cfu; see also ≥48 h in Figure 1C). (C) Killing curve of *S.* Tm from the early logarithmic growth phase of a culture (37°C in LB). Bacteria were incubated with ciprofloxacin (10 µg/ml), and the surviving bacteria were quantified by plating on LB agar (no antibiotics). The killing curve is biphasic as expected if the bacteria form a (large) susceptible and a (smaller) tolerant subpopulation.
(EPS)

Figure S3 **Tolerant** ***S.*** **Tm cells in the cecal patch and the cecum mucosa.** Mice were infected as in Figure 1, and we analyzed pathogen loads in the cecal patch and the cecum gut tissue of the ciprofloxacin-treated mice. *p*, significant differences of cfu loads as compared to the detection limit (10 cfu per tissue; strippled line).
(EPS)

Figure S4 **Tolerant** ***S.*** **Tm in cLN or the spleens of mice treated after days 1, 2, or 3.** C57BL/6 mice were infected for 1, 2, or 3 d with *S.* Tm (5×10^7^ cfu by gavage) and ciprofloxacin treatment (2×62 mg/kg/d) was started 2 d before the end of the experiment. Cfus in the cLN (A) and the spleen (B) were determined by plating. Each symbol represents one mouse. The *S.* Tm population in the cLN displays a higher fraction of tolerant cells than the bacteria lodged in the spleen. Dashed line, detection limit. *p*, significant differences of cfu loads compared to animals at the onset of ciprofloxacin treatment.
(EPS)

Figure S5 **Cellular penetration of ciprofloxacin.** HeLa Kyoto cells were infected with *S.* Tm pRFP ([23]; MOI, 1, 20′, 37°C, 5% CO_2_), treated with 150 µg/ml ciprofloxacin and the fluid phase marker 10-kD Dextran-Cy5 (1 mg/ml), and imaged by confocal fluorescence microscopy (Cipro, excitiation, 365; emission, 445/450). Fluorescence profiles show that ciprofloxacin (but not Dextran-Cy5) was penetrating efficiently into all intracellular compartments of the host cell within minutes after the beginning of the experiment.
(EPS)

Figure S6 **The virulence of** ***S.*** **Tm re-isolated from the cLN of ciprofloxacin-treated mice remains unaltered.** Five re-isolated SB300 strains (*S.* Tm SL1344) were competed against their parental strain carrying a neutral antibiotic marker (*marT*::cat; [53]). Competitive index (CI) after 2 d of i.p. infection of naïve C57BL/6 mice (*n* = 4–5) shows no significant fitness phenotype of re-isolated clones. In conclusion, even though *S.* typhimurium mutants conferring increased resistance to the antibiotic and altered gene expression characteristics have been isolated occasionally from humans or veterinary sources (Piddock, 1990), our data exclude that such mutants have occurred in our experiments. The antibiotic tolerance was attributable to a phenotypic adaptation. *p*, significant difference of CI from 1. n.s., not significant.
(EPS)

Figure S7 **Ciprofloxacin treatment cannot clear** ***S.*** **Tm from the cLN of 129SvEv mice.** This mouse line is *nramp*-positive and therefore resistant to deadly systemic *S.* Typhimurium infection. Two groups of 129SvEv mice were infected with *S.* Tm (5×10^7^ cfu by gavage). One group was analyzed at day 1 p.i. The second group was treated with ciprofloxacin (2×62 mg/kg/d) after day 1 p.i., and we analyzed cfus in the cLN at 3 d p.i. Each symbol represents one mouse. Dashed line, detection limit. *p*, nonparametric statistical analysis compared to 1 d p.i..
(EPS)

Figure S8 ***S.*** **Tm strain ATCC14028 also yields ciprofloxacin-tolerant cells in vivo.** C57BL/6 mice were infected with ATCC14028 and treated (or not) with ciprofloxacin, as described in Figure 1. Pathogen loads in the cecum lumen, the spleen, and the cLN were quantified by plating at the indicated time points. Dashed line, detection limit. *p*, nonparametric statistical analysis. n.s., not significant.
(EPS)

Figure S9 **Susceptibility to ciprofloxacin at different phases of the growth curve in vitro.** Samples of *S.* Tm grown at 37°C in LB culture were taken during different growth phases, and susceptibility to ciprofloxacin (10 µg/ml) was tested. Survival after 1 h of incubation was quantified by determining the number of cfus on LB agar plates (no antibiotics). The results were plotted against the growth rate. Samples taken during early log-phase were most susceptible to antibiotic killing (a and b), whereas slow growing bacteria were not (d). Plots show the mean of three independent experiments.
(EPS)

Figure S10 **Total cfu plots for the experiment shown in** **Figure 2** **.**
(EPS)

Figure S11 **Intracellular** ***S.*** **Tm display a high fraction of ciprofloxacin-tolerant bacteria.** *S.* Tm was grown in LB to OD 0.2 and used to inoculate BMDC, which had been seeded into a 24-well dish. One hour after infection, extracellular bacteria were removed by washing, and the cells were placed for 13 h in tissue culture medium harboring 200 µg/ml gentamycin. Ciprofloxacin (10 µg/ml) was added for the indicated times and the remaining bacteria were quantified by plating (closed symbols). Open symbols, bacteria of the inoculum were incubated directly with ciprofloxacin, and we quantified the surviving bacteria by plating. Intracellular bacteria show a larger persistent subpopulation.
(EPS)

Figure S12 **Experimental strategy for analyzing the population dynamical parameters of the ciproflocaxin-tolerant** ***S.*** **Tm in the cLN.** (A) Analysis of the population dynamics during the first 24 h p.i. Mice were infected with a 20∶1 mixture of *S.* Tm and *S.* Tm^WITS^ for 24 h. The cLN was aseptically removed and homogenized. Half of the cLN was inoculated into a kanamycin-containing LB culture to enrich for *S.* Tm^WITS^ for later rtqPCR, and the rest was plated on streptomycin or kanamycin plates for cfu determination (see Materials and Methods). (B) To analyze population dynamics under ciprofloxacin, mice were infected for 24 h with a 20∶1 mixture of *S.* Tm and *S.* Tm^WITS^ and ciprofloxacin treatment (2×62 mg/kg/d by gavage) was started at 24 h p.i.. After 72 h, 120 h, or 240 h, mice were sacrificed and *S.* Tm^WITS^ presence and abundance was determined for each time point. Using iterations of simulation between two adjacent time points (24–72 h; 72–120 h; 120–240 h), the model was extended to day 10 (see Materials and Methods).
(EPS)

Figure S13 **rtPCR analysis of pro-inflammatory gene expression.** Real time PCR analysis of cLN of C57BL/6 mice (3–7 mice per group) infected (or not) with *S.* Tm for 24 h and sacrificed at 2 d postinfection with or without ciprofloxacin treatment (2×62 mg/kg/d) and with or without treatment with LPS (5 µg of LPS, intraperitoneally 2 h before sacrifice), as indicated. Quantitative real-time PCR analysis of selected genes reveals a reduction of inflammatory status upon antibiotic treatment, which is enhanced after LPS injection. In particular, the expression of *cxcl2*, *ifng*, *s100a9*, and *lcn2* were reduced in ciprofloxacin-treated mice (compare black and gray bars). Interestingly, *tnf* (and possibly *il1a*) levels did not respond to ciprofloxacin treatment. The reasons for this remain to be analyzed. LPS treatment augmented the expression of *cxcl2*, *s100a9*, and *lcn2*. The data may suggest that different elements of the innate defense may respond with different kinetics. *p*, nonparametric statistical analysis (Mann–Whitney U-test). n.s., not significant.
(EPS)

Figure S14 **Fecal and spleen pathogen loads of mice treated with ciprofloxacin and LPS or CpG as shown in** **Figure 6** **.** C57BL/6 mice were infected for 1 d or 3 d with *S.* Tm (5×10^7^ cfu by gavage). Ciprofloxacin treatment (2×62 mg/kg/d by gavage) was started at day 1 p.i. and continued until the end of the experiment, as indicated. The indicated groups were treated with a single dose of LPS (5 µg intraperitoneally) or CpG (100 µg intraperitoneally) at day 2 p.i. Cfus in the spleens and the feces were analyzed by plating. Each symbol represents one mouse. Dashed line, detection limit. *p*, nonparametric statistical analysis (Mann–Whitney U-test). In the cLN, the LPS or CpG treatment alone does not significantly affect the *S.* Tm tissue load (Figure 6). In contrast, the spleen loads are reduced if CpG alone is applied (Figure S14). The same may hold true for the LPS treatment, if more mice were analyzed (not significant with five mice per group). In the gut lumen, LPS (but not CpG) significantly reduced pathogen loads. This may indicate that innate defenses may directly or indirectly augment pathogen elimination from these sites (but not in the cLN; see Figure 6) even in the absence of antibiotic treatment. The underlying mechanism remains to be elucidated.
(EPS)

Figure S15 **LPS can reduce loads of tolerant** ***S.*** **Tm in the cecal patch and the cecum mucosa.** C57BL/6 mice were infected for 3 d with *S.* Tm (5×10^7^ cfu by gavage). Ciprofloxacin treatment (2×62 mg/kg/d by gavage) was started at day 1 p.i. and continued until the end of the experiment. The indicated groups were treated with a single dose of LPS (5 µg intraperitoneally) at day 2 p.i. Cfus in the cecal patch and the cecum wall were analyzed by plating. Each symbol represents one mouse. Dashed line, detection limit. *p*, nonparametric statistical analysis (Mann–Whitney U-test). Cfus in the cecal patch and cecal wall of C57BL/6 mice 3 d postinfection with ciprofloxacin treatment (2×62 mg/kg/d) starting 1 d p.i. and injection of LPS (5 µg intreaperitoneally) after 1 d of ciprofloxacin treatment. Each symbol represents one mouse. Dashed lines, detection limits.
(EPS)

Figure S16 **LPS may enhance** ***S.*** **Tm elimination in vivo via an indirect mechanism (red symbols).** We generated bone marrow chimeric mice by lethally irradiating C57BL/6 (Ly5.1) mice and reconstituting with 50% *tlr4* ^−/−^ (Ly5.2) and 50% wt bone marrow (Ly5.1). After 6 wk, the reconstitution efficiency of the BMDCs (including the cDC) was tested. The mice harbored 50%–70% *tlr4* ^−/−^ cells. These mice and nonchimeric C57BL/6 controls were infected for 3 d with *S.* Tm(pAM34) and treated with ciprofloxacin (2×62 mg/kg/d by gavage) starting at day 2 p.i. and with LPS (5 µg i.p.) at 48 h p.i. Total *S.* Tm loads (red closed symbols) and *S.* Tm(pAM34) (red open symbols) in the cLN were determined by plating. Dashed line, detection limit. *p*, nonparametric statistical analysis. n.s., not significant. The experiment had been performed in parallel with that shown in Figure 5. For comparison, we have replotted the data from Figure 5 (these data are shown in black). There was no significant difference between the wild-type control mice and the *tlr4* ^−/−^/wt-mixed bone marrow chimaeric animals. These data indicate that LPS can enhance the clearance of ciprofloxacin-tolerant bacteria from the cLN via an indirect (paracrine) mechanism. The molecular basis remains to be established.
(EPS)

Protocol S1 **R-package containing the WITS data analyzed, as well as the likelihood and simulation functions.** Because the simulation function depends on the R-package GillespieSSA, install this package first by executing ‘install.packages(“GillespieSSA”)’ in R. After downloading the .gz file (protocol S1, named kaiser14bp.gz), it can then be installed in R by executing ‘install.package(“<path to downloaded .gz file>”, repos = NULL, type = “source”)’. After installation, load the package by executing “library(“kaiser14pb”)”. Get started by calling the main manual page: ‘?kaiser14pb’.
(GZ) Click here for additional data file.

Table S1 **Analysis of the MIC.** Using a plating assay [57] we analyzed the concentration of ciproflocaxin required to detectably inhibit growth of the parental *S.* Tm strain SL1344, *S.* Tm strain ATCC14028 and five isolates of each strain re-isolated from the cLN of mice (after 2 d ciprofloxacin treatment). These data verify that phenotypic tolerance (not genetic resistance mutation) explains bacterial survival in the cLN. +, unaltered growth; −, diminished growth.
(XLS)

Table S2 **Raw data used for modeling in vivo growth and killing rates under ciprofloxacin treatment.** The table shows the raw data gained from mouse experiments, which were used to fit the mathematical model. Each row shows the absolute number of *S.* Tm carrying a specific WITS-tag from one mouse cLN, total cLN cfu (tagged and untagged *S.* Tm), and number of days of infection. Ciprofloxacin treatment (2×62 mg/kg/d by gavage) was started day 1 p.i. for all mice. This dataset is also contained in the R-package provided as Protocol S1.
(XLS)

Text S1 **Documentation of the R-package kaiser14pb in .pdf format.**
(PDF)

## Abbreviations

BMDC: bone-marrow–derived dendritic cell
*c*: rate of clearance
cDC: classical dendritic cell (CD103^+^CX_3_CR1^−^CD11c^+^)
cfu: colony forming units
cLN: cecum draining lymph node
DTR: diphtheria toxin receptor
DTX: diphtheria toxin
FACS: fluorescence activated cell scanning
iDCs: interstitial dendritic cells (CD103^−^CX_3_CR1^+^)
i.p.: intra-peritoneally
LPS: lipopolysaccharide
MIC: minimal inhibitory concentration
*r*: rate of replication
*S*. Tm: *Salmonella enterica* subspecies 1 serovar Typhimurium
*μ*: rate of immigration
WITS: wild-type isogenic tagged strains
