# Supplementary material for: Cecum Lymph Node Dendritic Cells Harbor Slow-Growing Bacteria Phenotypically Tolerant to Antibiotic Treatment
Source: PLoS Biol. 2014 Feb 18;12(2):e1001793. doi: 10.1371/journal.pbio.1001793 (PMC3928039; doi:10.1371/journal.pbio.1001793)
Supplement: Text S1 — Documentation of the R-package kaiser14pb in .pdf format. (PDF) [file pbio.1001793.s020.pdf]

Package ‘kaiser14pb’

December 19, 2013

Type Package

Title Supplementary online material accompanying Kaiser et al, PLoS Biol (2014)

Version 1.0

Date 2013-12-16

Author Roland R Regoes

Maintainer The package is not maintained, but questions can be sent to
Roland R Regoes <roland.regoes@env.ethz.ch>

Depends GillespieSSA

Description This package contains the datasets, likelihoods and simulation functions used in Kaiser et al, PLoS Biol (2014)

License GPL (>= 2)

LazyData yes

KeepSource yes

NeedsCompilation no

R topics documented:

kaiser14pb-package 2
bdi.sim 3
fit.functions.kaiser14pb 4
kaiser14pb.data 6
kaiser14pb.MLEs 7
likelihoods.kaiser14pb 8
pgf.kaiser14pb 10
sim.treat 12

Index **14**

`kaiser14pb-package` *Supplementary online material accompanying Kaiser et al, PLoS Biol (2014)*

Description

This package contains the WITS data, likelihood and simulation functions used in Kaiser et al, PLoS Biol (2014).

Details

Package:	kaiser14pb
Type:	Package
Version:	1.0
Date:	2013-12-16
License:	GPL (>= 2)

This material is provided mostly for the sake of transparency. The functions were developed for the specific research questions and datasets presented in Kaiser et al PLoS Biol (2014) and Kaiser et al, PLoS Path (2013). They may not work beyond this restricted field of application.

We tried to document the functions as well as we could. However, documentation is only rudimentary, and will remain so because the package will not be updated.

Some of the experimental data presented and analyzed in Kaiser et al, PLoS Biol (2014) are included in this package (see `kaiser14pb.data`).

For a summary of the probability generating functions and likelihoods see `pgf.kaiser14pb` and `likelihoods.kaiser14pb`, respectively.

The convenience routines described in `fit.functions.kaiser14pb` allow you to maximize the likelihoods. The parameter estimates we obtained by fitting the models are stored in the dataset `kaiser14pb.MLEs` contained in this package.

See `bdi.sim` and `sim.treat` for documentation of the simulation functions. These functions rely on `ssa` in the package GillespieSSA.

Author(s)

Roland R Regoes <roland.regoes@env.ethz.ch>

The package is not maintained. It is provided as supplementary online material to the paper Kaiser et al PLoS Biol (2014).

References

Kaiser P, Regoes RR, Dolowschiak T, Wotzka S, Lengefeld J, Slack E, Grant AJ, Ackermann M and Hardt WD (2014). Cecum lymph node dendritic cells harbor slow-growing bacteria phenotypically tolerant to antibiotic treatment. PLoS Biol.

Kaiser P, Slack E, Grant AJ, Hardt WD, Regoes RR (2013). Lymph node colonization dynamics after oral salmonella typhimurium infection in mice. PLoS Path.

See Also

`kaiser14pb.data`, `pgf.kaiser14pb`, `likelihoods.kaiser14pb`, `fit.functions.kaiser14pb`, `kaiser14pb.MLEs`, `bdi.sim`, `sim.treat`.

Examples

```
# Compact display of the WITS data included in this package:
str(kaiser14pb.data)

# Estimate parameters r and muW for the from the untreated mice
# sacrificed one day after infection:
fit.function.c0()
```

`bdi.sim` *Stochastic birth-death-imigration model simulation*

Description

Function simulating the stochastic birth-death model with immigration used in Kaiser et al, PLoS Biol (2014).

Usage

```
bdi.sim(parms = c(r = 3, c = 0, mu = 1),
        time.final = 1, M0 = 0,
        output.data = FALSE)
```

Arguments

`parms`	Named vector of the form `c(r = 3, c = 0, mu = 1)` containing the three parameters of the birth-death-imigration model.
`time.final`	Scalar. The time until the simulation should run.
`M0`	Integer. The initial number of individuals in the populations (bacteria in the lymph node).
`output.data`	Logical indicating if the entire simulation is returned.

Details

The function requires the package GillespieSSA.

The function does not simulate properly for `c > 0` and `mu > 0`. If the simulation reaches a population size of 0, it always terminates. It never restarts, although it sometimes should if `mu > 0`.

This function is used in `sim.treat`.

Value

If `output.data` is set to `FALSE` a positive integer is returned with the population size at around `time.final`.

Otherwise `bdi.sim` returns a list with two elements:

`final`	a positive integer is returned with the population size at around `time.final`
`data`	A dataframe with two columns, one for time `t` and the second for population size `M`.

Author(s)

Roland R Regoes <roland.regoes@env.ethz.ch>

The package is not maintained. It is provided as supplementary online material to the paper Kaiser et al PLoS Biol (2014).

References

Kaiser P, Regoes RR, Dolowschiak T, Wotzka S, Lengefeld J, Slack E, Grant AJ, Ackermann M and Hardt WD (2014). Cecum lymph node dendritic cells harbor slow-growing bacteria phenotypically tolerant to antibiotic treatment. PLoS Biol.

See Also

`kaiser14pb`, `kaiser14pb.data`, `pgf.kaiser14pb`, `likelihoods.kaiser14pb`, `fit.functions.kaiser14pb`, `kaiser14pb.MLEs`, `sim.treat`.

Examples

```
# To generate one instance of the birth-death immigration model:
bdi.sim(parms = c(r = 3, c = 0, mu = 1),
        time.final = 1, M0 = 0)
```

`fit.functions.kaiser14pb`

Fit functions

Description

Fit functions maximizing the likelihoods in the paper by Kaiser et al, PLoS Biol (2014).

Usage

```
fit.function.c0(data = subset(kaiser14pb.data, mouse.type=="wt"),
                pgf = NULL, output.sd = T)

fit.function.cfix(data = subset(kaiser14pb.data, mouse.type=="wt"),
                  cfix = 0, pgf = NULL, output.sd = T)

fit.function.muG0(data = subset(kaiser14pb.data, mouse.type=="wt+Cipro" & day==3),
                  pgf = pgf.treat.d3, output.sd = T)

fit.function.pbs(data = subset(kaiser14pb.data, mouse.type=="wt+PBS"),
                 pgf = pgf.pbs, output.sd = T)
```

Arguments

`data`	Dataframe of the form `kaiser14pb.data`.
`pgf`	Function encoding a probability generating function of the form `pgf.kaiser14pb`. Only used in `fit.function.muG0` and `fit.function.pbs`.
`cfix`	Non-negative scalar. This passes a fixed value for c to `fit.function.cfix`. For the default `cfix = 0`, `fit.function.cfix` is equivalent to `fit.function.c0`.
`output.sd`	Logical indicating if standard deviations of parameter estimates should be returned.

Details

These are convenience functions, wrapping around `optim`.

`fit.function.c0` fits assuming an initially empty lymph node and `c=0`.

`fit.function.cfix` fits assuming an initially empty lymph node and `c=cfix`.

`fit.function.muG0` fits assuming an initial distribution of bacterial populations size in the lymph node specified by `pgf` and `muG=0`.

`fit.function.pbs` fits assuming an initial distribution of bacterial populations size in the lymph node specified by `pgf` and `c=0`.

Value

A list with components:

`pars`	Vector with the maximum likelihood estimates.
`sd`	Vector with the standard deviations of the maximum likelihood estimates.
`ll`	Scalar with the value of the log likelihood at its maximum.
`convergence`	`optim`'s convergence output.
`fit.message`	`optim`'s message output.

Author(s)

Roland R Regoes <roland.regoes@env.ethz.ch>

The package is not maintained. It is provided as supplementary online material to the paper Kaiser et al PLoS Biol (2014).

References

Kaiser P, Regoes RR, Dolowschiak T, Wotzka S, Lengefeld J, Slack E, Grant AJ, Ackermann M and Hardt WD (2014). Cecum lymph node dendritic cells harbor slow-growing bacteria phenotypically tolerant to antibiotic treatment. PLoS Biol.

Kaiser P, Slack E, Grant AJ, Hardt WD, Regoes RR (2013). Lymph node colonization dynamics after oral salmonella typhimurium infection in mice. PLoS Path.

See Also

`kaiser14pb`, `kaiser14pb.data`, `pgf.kaiser14pb`, `likelihoods.kaiser14pb`, `kaiser14pb.MLEs`, `bdi.sim`, `sim.treat`.

Examples

```
# This fits the day 1 WITS data (takes a few seconds):
fit.function.c0()
```

`kaiser14pb.data` *WITS data*

Description

WITS data presented and analyzed in Kaiser et al, PLoS Biol (2014).

Usage

```
data(kaiser14pb.data)
```

Format

A data frame with 231 observations on the following 8 variables.

- `day` a numeric vector with the day after inoculation, at which the mouse was sacrificed and bacterial counts measured
- `mouse.type` a factor with levels `wt`, `wt+Cipro`, and `wt+PBS`, indicating the genotype and treatment the mouse received.
- `mouse` a factor with many levels; this is a meaningless mouse ID
- `salmonella.strain` a factor with only one level `SB300`; this is the Salmonella Typhimuroim strain we used
- `total` a numeric vector; gives the total number of bacteria in the lymph node (measures as CFU)

`WITS.dilution` a numeric vector with the fractions of each WITS in the inoculum

`WITS` a factor with levels `1 11 13 17 19 2 21`; this is a "WITS ID" (which is unfortunately an integer); there are seven such IDs

`number` a numeric vector containing the number of each WITS recovered from the lymph node at day `day`

Source

Kaiser P, Regoes RR, Dolowschiak T, Wotzka S, Lengefeld J, Slack E, Grant AJ, Ackermann M and Hardt WD (2014). Cecum lymph node dendritic cells harbor slow-growing bacteria phenotypically tolerant to antibiotic treatment. PLoS Biol.

See Also

`kaiser14pb`, `pgf.kaiser14pb`, `likelihoods.kaiser14pb`, `fit.functions.kaiser14pb`, `kaiser14pb.MLEs`, `bdi.sim`, `sim.treat`.

Examples

```
# Load the data (although this should not be necessary,
# as the data are loaded with the package):
data(kaiser14pb.data)

# Get a rough idea of the data:
str(kaiser14pb.data)
```

`kaiser14pb.MLEs` *Maximum likelihood parameter estimates*

Description

Maximum likelihood parameter estimates of the stochastic birth-death-immigration model published in Kaiser et al, PLoS Biol (2014).

Usage

```
data(kaiser14pb.MLEs)
```

Format

A data frame with 5 observations on the following 6 variables.

`dataset` a factor containing the "experiment ID":

`SB300`	untreated	sacrificed day 1
`SB300.cipro.d3`	ciprofloxacin treated (after day 1)	sacrificed day 3
`SB300.cipro.d5`	ciprofloxacin treated (after day 1)	sacrificed day 5
`SB300.cipro.d10`	ciprofloxacin treated (after day 1)	sacrificed day 10
`SB300.pbs`	PBS treated (after day 1)	sacrificed day 3

ll A scalar with the value of the log-likelihood at its maximum

r maximum likelihood estimate of replication rate r

c maximum likelihood estimate of clearance rate c

muW maximum likelihood estimate of WITS immigration rate muW

mu.total Immigration rate of total bacterial population corresponding to the WITS immigration rate muW. This is simply the muW times the entry under WITS.dilution in kaiser14pb.data

Details

This dataset is included because some of the probability generating functions (pgf.kaiser14pb) rely these estimates. In particular, pgf.treat.d3, pgf.treat.d5, pgf.treat.d10, and pgf.pbs, which characterize the dynamics after the first day of infection, internally evaluate pgf.salmonella at the maximum likelihood estimates for the first day of infection.

In addition, the simulation function sim.treat uses the maximum likelihood estimates as default parameters.

Source

Kaiser P, Regoes RR, Dolowschiak T, Wotzka S, Lengefeld J, Slack E, Grant AJ, Ackermann M and Hardt WD (2014). Cecum lymph node dendritic cells harbor slow-growing bacteria phenotypically tolerant to antibiotic treatment. PLoS Biol.

See Also

kaiser14pb, kaiser14pb.data, pgf.kaiser14pb, likelihoods.kaiser14pb, fit.functions.kaiser14pb, bdi.sim, sim.treat.

Examples

```
# Look at the estimates:
kaiser14pb.MLEs
```

---

likelihoods.kaiser14pb *Likelihood functions*

Description

Likelihood functions used in Kaiser et al, PLoS Biol (2014).

Usage

```
ll.salmonella.mdl.roger(parms, data)

pk.log(parms, dataline)

ll.salmonella.mdl.fourier(parms, data,
                          prob.gen.fct = pgf.treat.d3,
                          N = 2^14)
```

Arguments

parms	Named vector of length three containing model parameter at which to evaluate the likelihood. It has to have the structure `c(r=3,c=0,muG=1)` (with exactly these names!). The values for each parameter need to be non-negative.
data	A dataframe with the structure of `kaiser14pb.data`. The dataframe requires two columns, named `day` and `number`. Entries under `day` have to be positive scalars, `number` entries have to be non-negative integers.
dataline	A data.frame with a single row. The structure has to be similar to `data`, and by extension to `kaiser14pb.data`.
prob.gen.fct	A function with the probability generation function describing the stochastic process. Can be any function described in `pgf.kaiser14pb`.
N	Large integer, best a power of 2. This number is used internally by `ll.salmonella.mdl.fourier` and determines the accuracy of the likelihood value computed.

Details

`ll.salmonella.mdl.roger` calculates the likelihood using an analytical expression for the state probabilities. This expression was obtained with the help of Roger Kouyos (which explains the name of the function).

`ll.salmonella.mdl.fourier` uses the Fast Fourier Transform `fft` to numerically compute the state probabilities from the probability function. That is why it needs `prob.gen.fct` as an input.

Value

List with two elements:

ll	Scalar containing the log-likelihood value
gr	A vector of length 3 gradient of the log-likelihood with respect to the three model parameters r, c, muG. This is only calculated by `ll.salmonella.mdl.roger`. `ll.salmonella.mdl.fourier` returns `c(NA,NA,NA)`.

Author(s)

Roland Regoes <roland.regoes@env.ethz.ch>

The package is not maintained. It is provided as supplementary online material to the paper Kaiser et al PLoS Biol (2014).

References

Kaiser P, Regoes RR, Dolowschiak T, Wotzka S, Lengefeld J, Slack E, Grant AJ, Ackermann M and Hardt WD (2014). Cecum lymph node dendritic cells harbor slow-growing bacteria phenotypically tolerant to antibiotic treatment. PLoS Biol.

Kaiser P, Slack E, Grant AJ, Hardt WD, Regoes RR (2013). Lymph node colonization dynamics after oral salmonella typhimurium infection in mice. PLoS Path.

See Also

kaiser14pb, kaiser14pb.data, pgf.kaiser14pb, fit.functions.kaiser14pb, kaiser14pb.MLEs, bdi.sim, sim.treat.

Examples

```
# Lets evaluate these functions at the MLEs:
ll.salmonella.mdl.roger(parms=c(r=2.82, c=0, muG=2.13),
                        data=subset(kaiser14pb.data, mouse.type=="wt"))

ll.salmonella.mdl.fourier(parms=c(r=4.59, c=5.04, muG=0),
                          data=subset(kaiser14pb.data,
                                      mouse.type=="wt+Cipro" & day==3))
```

---

pgf.kaiser14pb *Probability generating functions*

Description

Probability generating functions used in Kaiser et al, PLoS Biol (2014).

Usage

```
pgf.salmonella(parms = c(r = 3, c = 0, muG = 1), t = 1, s)

pgf.treat.d3(parms = c(r = 3, c = 3.5, muG = 0),
             t = 3, s)

pgf.treat.d5(parms = c(r = 3, c = 3.5, muG = 0),
             t = 5, s)

pgf.treat.d10(parms = c(r = 3, c = 3.5, muG = 0),
              t = 10, s)

pgf.pbs(parms = c(r = 3, c = 0, muG = 1),
        t = 3, s)
```

Arguments

parms	Named vector containing the parameters at which the pgf should be evaluated. The elements should have names "r", "c", and "muG"
t	Scalar, indicating the time in days.
s	Scalar between 0 and 1. Dummy variable of the probability generating function.

Details

Some of these functions are used to calculate the likelihoods (see likelihoods.kaiser14pb).

pgf.salmonella is the probability generating function for a process starting with an empty lymph node.

pgf.treat.d3 is the probability generating function for a process starting with some bacteria in the lymph node. The distribution of the number of bacteria is given by pgf.salmonella evaluated at the maximum likelihood estimates for the parameters r, and muG obtained during the first day. The function is only valid assuming muG=0 during treatment.

pgf.treat.d5 and pgf.treat.d10 give the probability generating functions at later days. They rely on the pgf from the previous periods, i.e. pgf.treat.d3 and pgf.treat.d5, respectively. These functions are also only valid assuming muG=0 during treatment.

pgf.pbs encodes the probability generating function for a process starting with some bacteria in the lymph node, and does not require muG=0.

Value

Scalar value of the probability generating function (which is always between 0 and 1).

Author(s)

Roland Regoes <roland.regoes@env.ethz.ch>

The package is not maintained. It is provided as supplementary online material to the paper Kaiser et al PLoS Biol (2014).

References

Kaiser P, Regoes RR, Dolowschiak T, Wotzka S, Lengefeld J, Slack E, Grant AJ, Ackermann M and Hardt WD (2014). Cecum lymph node dendritic cells harbor slow-growing bacteria phenotypically tolerant to antibiotic treatment. PLoS Biol.

Kaiser P, Slack E, Grant AJ, Hardt WD, Regoes RR (2013). Lymph node colonization dynamics after oral salmonella typhimurium infection in mice. PLoS Path.

See Also

kaiser14pb, kaiser14pb.data, likelihoods.kaiser14pb, fit.functions.kaiser14pb, kaiser14pb.MLEs, bdi.sim, sim.treat.

Examples

```
# Execute the functions at default parameters:
pgf.salmonella(t = 1, s = 0)
pgf.treat.d3(t = 3, s = 0)
pgf.treat.d5(t = 5, s = 0)
pgf.treat.d10(t = 10, s = 0)
pgf.pbs(t = 3, s = 0)

# They all should give 1 for s=1:
pgf.salmonella(t = 1, s = 1)
pgf.treat.d10(t = 10, s = 1)
pgf.pbs(t = 3, s = 1)
```

---

sim.treat   *Simulating the colonization of the cecal lymph node by Salmonella under antibiotic treatment.*

Description

Function simulating the colonization dynamics of Salmonella typhimurium in the cecal lymph node under treatment used in Kaiser et al, PLoS Biol (2014). It can be used to produce Figure 4B of the paper.

Usage

```
sim.treat(parms.d1 = c(r = kaiser14pb.MLEs[kaiser14pb.MLEs$dataset == "SB300","r"],
                       c = 0,
                       mu= kaiser14pb.MLEs[kaiser14pb.MLEs$dataset == "SB300","mu.total"]),
          parms.d3 = c(r = kaiser14pb.MLEs[kaiser14pb.MLEs$dataset == "SB300.cipro.d3","r"],
                       c = kaiser14pb.MLEs[kaiser14pb.MLEs$dataset == "SB300.cipro.d3","c"],
                       mu= 0),
          parms.d5 = c(r = kaiser14pb.MLEs[kaiser14pb.MLEs$dataset == "SB300.cipro.d5","r"],
                       c = kaiser14pb.MLEs[kaiser14pb.MLEs$dataset == "SB300.cipro.d5","c"],
                       mu= 0),
          parms.d10= c(r = kaiser14pb.MLEs[kaiser14pb.MLEs$dataset == "SB300.cipro.d10","r"],
                       c = kaiser14pb.MLEs[kaiser14pb.MLEs$dataset == "SB300.cipro.d10","c"],
                       mu= 0),
          output.data = TRUE)
```

Arguments

parms.d1	Named vector of the form c(r=3, c=0, mu=300) containing the parameters characterizing the colonization of the cecal lymph node during the first day after inoculation. Defaults to the maximum likelihood estimates stored in kaiser14pb.data.
parms.d3	Named vector of the form c(r=3, c=0, mu=300) containing the parameters characterizing the colonization of the cecal lymph node between day 1 and 3 of infection. Defaults to the maximum likelihood estimates stored in kaiser14pb.data.

`parms.d5` Named vector of the form `c(r=3, c=0, mu=300)` containing the parameters characterizing the colonization of the cecal lymph node between day 3 and 5 of infection. Defaults to the maximum likelihood estimates stored in `kaiser14pb.data`.

`parms.d10` Named vector of the form `c(r=3, c=0, mu=300)` containing the parameters characterizing the colonization of the cecal lymph node between day 5 and 10 of infection. Defaults to the maximum likelihood estimates stored in `kaiser14pb.data`.

`output.data` Logical indicating if the the population size throughout the simulation time should be returned. If set to `FALSE` only the population sizes at day 1, 3, 5, and 10 are returned.

Details

This convenience function simply concatenates simulations run piecewise using `bdi.sim` with the maximum likelihood estimates for r, c, and mu. It simulates the first ten days of infection. With default parameters it simulates the colonization of the lymph node in mice that are treated 1 day after infection.

Value

If `output.data` is set to `TRUE` a dataframe is returned with two columns, one for time `t` and the second for population size `M`.

If `output.data` is set to `FALSE` a named integer vector is returned that contains the population sizes at day 1, 3, 5, and 10.

Author(s)

Roland R Regoes <roland.regoes@env.ethz.ch>

The package is not maintained. It is provided as supplementary online material to the paper Kaiser et al PLoS Biol (2014).

References

Kaiser P, Regoes RR, Dolowschiak T, Wotzka S, Lengefeld J, Slack E, Grant AJ, Ackermann M and Hardt WD (2014). Cecum lymph node dendritic cells harbor slow-growing bacteria phenotypically tolerant to antibiotic treatment. PLoS Biol.

See Also

`kaiser14pb`, `kaiser14pb.data`, `pgf.kaiser14pb`, `likelihoods.kaiser14pb`, `fit.functions.kaiser14pb`, `kaiser14pb.MLEs`, `bdi.sim`.

Examples

```
# To obtain a plot similar to Figure 4B in Kaiser et al,
# PLoS Biol (2014) execute (simulation takes approximately 5 sec):
st1 <- sim.treat()
plot(st1$t[-1], st1$M[-1],
     xlab="Days", ylab="Bacteria in cLN (CFU)",
     type="l", log="y")
```

Index

∗Topic **kaiser14pb**
kaiser14pb-package, 2

bdi.sim, 2, 3, 3, 6–8, 10, 11, 13

fft, 9
fit.function.c0 (fit.functions.kaiser14pb), 4
fit.function.cfix (fit.functions.kaiser14pb), 4
fit.function.muG0 (fit.functions.kaiser14pb), 4
fit.function.pbs (fit.functions.kaiser14pb), 4
fit.functions.kaiser14pb, 2–4, 4, 7, 8, 10, 11, 13

kaiser14pb, 4, 6–8, 10, 11, 13
kaiser14pb (kaiser14pb-package), 2
kaiser14pb-package, 2
kaiser14pb.data, 2–6, 6, 8–13
kaiser14pb.MLEs, 2–4, 6, 7, 7, 10, 11, 13

likelihoods.kaiser14pb, 2–4, 6–8, 8, 11, 13
ll.salmonella.mdl.fourier (likelihoods.kaiser14pb), 8
ll.salmonella.mdl.roger (likelihoods.kaiser14pb), 8

optim, 5

pgf.kaiser14pb, 2–10, 10, 13
pgf.pbs, 8
pgf.pbs (pgf.kaiser14pb), 10
pgf.salmonella, 8
pgf.salmonella (pgf.kaiser14pb), 10
pgf.treat.d10, 8
pgf.treat.d10 (pgf.kaiser14pb), 10
pgf.treat.d3, 8
pgf.treat.d3 (pgf.kaiser14pb), 10
pgf.treat.d5, 8
pgf.treat.d5 (pgf.kaiser14pb), 10
pk.log (likelihoods.kaiser14pb), 8

sim.treat, 2–4, 6–8, 10, 11, 12
ssa, 2
